# Calcineurin regulates aldosterone production via dephosphorylation of NFATC4

**DOI:** 10.1172/jci.insight.157027

**Published:** 2023-07-24

**Authors:** Mesut Berber, Sining Leng, Agnieszka Wengi, Denise V. Winter, Alex Odermatt, Felix Beuschlein, Johannes Loffing, David T. Breault, David Penton

**Affiliations:** 1 Institute of Anatomy, University of Zurich, Switzerland.; 2 Swiss National Centre for Competence in Research “Kidney Control of Homeostasis” (NCCR Kidney.CH), Zurich, Switzerland.; 3 Department of Pediatrics, Harvard Medical School, Boston Children’s Hospital, Boston, Massachusetts, USA.; 4 Division of Endocrinology, Boston Children’s Hospital, Boston, Massachusetts, USA.; 5 Swiss Centre for Applied Human Toxicology and Division of Molecular and Systems Toxicology, Department of Pharmaceutical Sciences, University of Basel, Basel, Switzerland.; 6 Department of Endocrinology, Diabetology and Clinical Nutrition, University Hospital Zurich and University of Zurich, Zurich, Switzerland.; 7 Medizinische Klinik und Poliklinik IV, Klinikum der Universität München, Munich, Germany.; 8 Harvard Stem Cell Institute, Cambridge, Massachusetts, USA.

**Keywords:** Endocrinology, Metabolism, Phosphoprotein phosphatases, Signal transduction

## Abstract

The mineralocorticoid aldosterone, secreted by the adrenal zona glomerulosa (ZG), is critical for life, maintaining ion homeostasis and blood pressure. Therapeutic inhibition of protein phosphatase 3 (calcineurin, Cn) results in inappropriately low plasma aldosterone levels despite concomitant hyperkalemia and hyperreninemia. We tested the hypothesis that Cn participates in the signal transduction pathway regulating aldosterone synthesis. Inhibition of Cn with tacrolimus abolished the potassium-stimulated (K^+^-stimulated) expression of aldosterone synthase, encoded by *CYP11B2*, in the NCI-H295R human adrenocortical cell line as well as ex vivo in mouse and human adrenal tissue. ZG-specific deletion of the regulatory Cn subunit CnB1 diminished *Cyp11b2* expression in vivo and disrupted K^+^-mediated aldosterone synthesis. Phosphoproteomics analysis identified nuclear factor of activated T cells, cytoplasmic 4 (NFATC4), as a target for Cn-mediated dephosphorylation. Deletion of *NFATC4* impaired K^+^-dependent stimulation of *CYP11B2* expression and aldosterone production while expression of a constitutively active form of *NFATC4* increased expression of *CYP11B2* in NCI-H295R cells. Chromatin immunoprecipitation revealed NFATC4 directly regulated *CYP11B2* expression. Thus, Cn controls aldosterone production via the Cn/NFATC4 pathway. Inhibition of Cn/NFATC4 signaling may explain low plasma aldosterone levels and hyperkalemia in patients treated with tacrolimus, and the Cn/NFATC4 pathway may provide novel molecular targets to treat primary aldosteronism.

## Graphical abstract

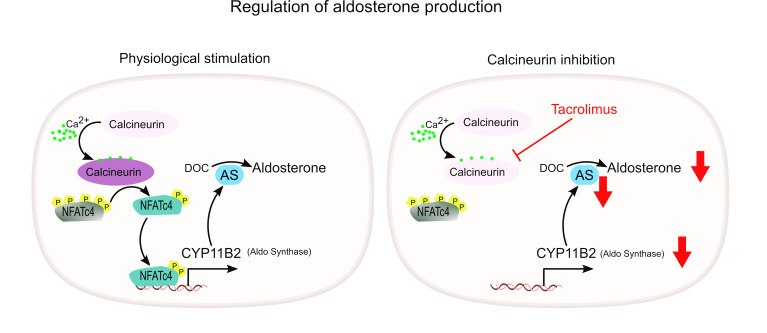

## Introduction

The mineralocorticoid aldosterone is secreted by zona glomerulosa (ZG) cells of the adrenal cortex and promotes sodium (Na^+^) reabsorption and potassium (K^+^) excretion in the kidneys; thereby, it is a key regulator of ion homeostasis and blood pressure (1). Indeed, primary aldosteronism (PA), characterized by an inappropriately high production of aldosterone, is the most frequent form of secondary hypertension, often accompanied by hypokalemia (2). Under physiological conditions, aldosterone secretion is stimulated by increased plasma concentrations of K^+^ or by hypovolemia via activation of the renin-angiotensin system (RAS) among other stimuli (3, 4).

Since steroid hormones are produced on demand, their synthesis is tightly controlled. The production of aldosterone is regulated at 2 levels, namely, the availability of its precursor and its synthesis. An acute increase in aldosterone production occurs following the phosphorylation and activation of the steroidogenic acute regulatory protein (StAR) in ZG cells. StAR is necessary to transport the steroid precursor cholesterol from the outer to the inner mitochondrial membrane, where aldosterone biosynthesis is initiated (5). Biosynthesis of aldosterone also requires expression of *CYP11B2*, the gene encoding aldosterone synthase, in the ZG (5).

Both angiotensin II (AngII) and K^+^ promote the depolarization of the ZG plasma membrane and activation of L and T type voltage-gated calcium (Ca^2+^) channels. The concomitant increase in intracellular Ca^2+^ subsequently activates calmodulin (CaM) (6–9). In addition, binding of AngII to its type 1 receptor activates phospholipase C and the production of phosphatidylinositol (3-5)-trisphosphate, which in turn promotes Ca^2+^ release from intracellular stores (5). Activated CaM binds to downstream proteins, including Ca^2+^/CaM-dependent protein kinases (CaMKs) (10, 11), which phosphorylate and activate the cAMP response element–binding protein (CREB), thereby promoting the expression of *CYP11B2* (5, 12–14). Additionally, both, K^+^ and AngII stimulate the expression of Nurr1 (encoded by *NR4A2*) and NGFIB (encoded by *NR4A1*) transcription factors that have been found to stimulate *CYP11B2* expression (15).

Although the CaMKs involved in the regulation of aldosterone production have been studied in detail (14, 16–18), other kinases, as well as the role of protein phosphatases, have attracted less attention. Interestingly, immunosuppressive therapy with calcineurin inhibitors (CnI) causes fludrocortisone-correctable hyperkalemia in up to 45% of patients (19–21). Thus, clinical evidence supports the hypothesis that CnI render ZG cells insensitive to changes in plasma K^+^ concentrations.

Calcineurin (Cn) is a serine/threonine heterodimeric phosphatase composed of a catalytic CnA subunit and a regulatory Ca^2+^ binding CnB subunit (22). The signaling pathway including Cn and the nuclear factor of activated T cells (NFATC) plays a pivotal role in a wide range of physiological and pathophysiological mechanisms, including those of immune, heart, nerve, and pancreatic cells (23–25). Cn dephosphorylates N-terminal residues in NFATC family members, leading to their nuclear translocation and concomitant transcriptional activation of target genes (26, 27). Whether the Cn/NFATC signaling pathway participates in the regulation of aldosterone production in ZG cells has not been studied in detail.

Previous in vitro studies suggested that Cn is involved in the AngII-stimulated production of aldosterone (28–30). Recently, tacrolimus (an inhibitor of Cn) was found to inhibit K^+^-induced stimulation of *CYP11B2* expression in NCI-H295R cells (31). Given the severe nephrotoxicity associated with CnI treatment (32), it has been difficult to confirm these observations, in vivo.

In this study, we investigate the regulation of *CYP11B2* expression and aldosterone production using in vitro, ex vivo, and in vivo approaches. We found that the Cn/NFATC4 pathway regulates the expression of *CYP11B2*. We discuss the implication of our findings for CnI-induced impairment of ion homeostasis and as a molecular target to treat overproduction of aldosterone.

## Results

### CnI blunt the stimulation of aldosterone synthase expression.

The adrenocortical cell line NCI-H295R (33) was used as a model to test the effect of protein phosphatase inhibitors (PPi) on the stimulation of *CYP11B2* expression. While most inhibitors had no effect, BCI (inhibitor of the dual-specificity protein phosphatase 1/6) and tacrolimus blunted the effect of K^+^ on *CYP11B2* expression as assessed by quantitative real-time PCR (qPCR) (Supplemental Figure 1A; supplemental material available online with this article; https://doi.org/10.1172/jci.insight.157027DS1). Moreover, tacrolimus inhibited the increase in *CYP11B2* expression elicited by AngII (Supplemental Figure 1B). Since NCI-H295R cells showed a higher sensitivity to high K^+^ than to AngII, we favored high K^+^ as a stimulation model throughout this study.

**Figure 1:**
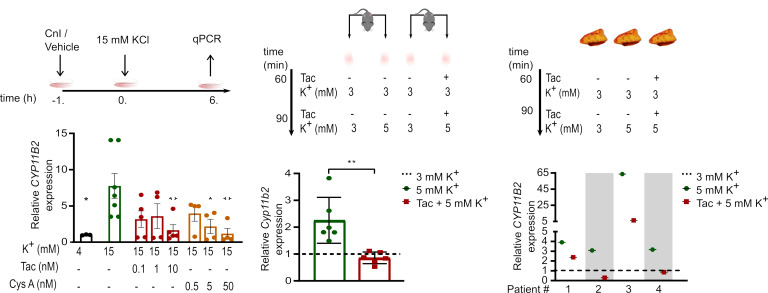
CnI blunt K^+^-stimulated aldosterone synthase gene expression. (**A**) NCI-H295R cells were stimulated with 15 mM K^+^ in the presence or absence of tacrolimus (0.1, 1, or 10 nM) or cyclosporine A (0.5, 5, or 50 nM) for 6 hours after preincubation with tacrolimus, cyclosporine, or vehicle for 1 hours (*n* = 3–7). The corresponding amount of NaCl was added to cells treated with 4mM K^+^ to compensate for changes in medium osmolality. *CYP11B2* expression levels were determined by qPCR. Statistical differences assessed by 1-way ANOVA with Dunnett’s multiple-comparison posttest. (*P < 0.05, **P < 0.01.) (**B**) Adrenal gland fragments from female C57BL/6 mice were perifused, ex vivo, with buffer containing vehicle or tacrolimus (Tac, 10 nM) for 60 minutes followed by buffer containing 5 mM K^+^ or 5 mM K^+^ + Tac for 90 minutes (*n* = 6). The contralateral adrenal from each mouse was perifused with 3 mM K^+^ as a control. *Cyp11b2* expression levels were determined by qPCR. Statistical differences assessed by unpaired 2-tailed Student’s *t* test. (**P < 0.01.) (**C**) Freshly resected human adrenal adenoma fragments were perifused, ex vivo, with buffer containing vehicle or Tac for 60 minutes followed by buffer containing 3 mM K^+^, 5 mM K^+^, or Tac + 5 mM K^+^ for 90 minutes (*n* = 4). The fragments perifused with buffer containing 3 mM K^+^ served as a control. *CYP11B2* expression levels were determined by qPCR. (Dashed line indicates the aldosterone synthase gene expression level of control mouse and human tissues.)

Upregulation of *CYP11B2* expression in NCI-H295R cells was inhibited in a dose-dependent manner by tacrolimus and cyclosporin A, 2 structurally and mechanistically distinct inhibitors of Cn (Figure 1A). To verify these observations using a more physiologically relevant model, female mouse adrenal fragments were incubated with media containing 3 mM K^+^ and stimulated with 5 mM K^+^. As shown in Figure 1B, stimulation with 5 mM K^+^ doubled the expression of *Cyp11b2* as assessed by qPCR. In adrenal fragments treated with tacrolimus, K^+^-dependent stimulation of *Cyp11b2* expression was completely blunted. Of note, adrenal pieces from male mice were not sensitive to stimulation with 5 mM K^+^ (Supplemental Figure 1C). Next, we extended the analysis using primary human adrenal adenoma fragments (Supplemental Table 1). Treatment with 5 mM K^+^ exhibited increased expression of *CYP11B2* as assessed by qPCR (Figure 1C). In agreement with the previously observed effect in NCI-H295R cells and mouse adrenal fragments, incubation of human adenoma fragments with tacrolimus dramatically impaired K^+^-dependent stimulation of *CYP11B2*. Together, our data indicate that Cn is crucial for K^+^-stimulated expression of aldosterone synthase.

### Genetic inactivation of Cn specifically in mouse ZG impairs aldosterone synthesis.

To exclude possible nonspecific effects of pharmacological modulators of Cn activity, we genetically deleted CnB1, the Ca^2+^-dependent regulatory subunit of Cn, specifically in ZG cells of mouse adrenal glands. CnB1 (encoded by *Ppp3r1*) is the only regulatory subunit of Cn expressed in the adrenal gland (Supplemental Figure 2A), and its deletion renders Cn inactive (34).

**Figure 2:**
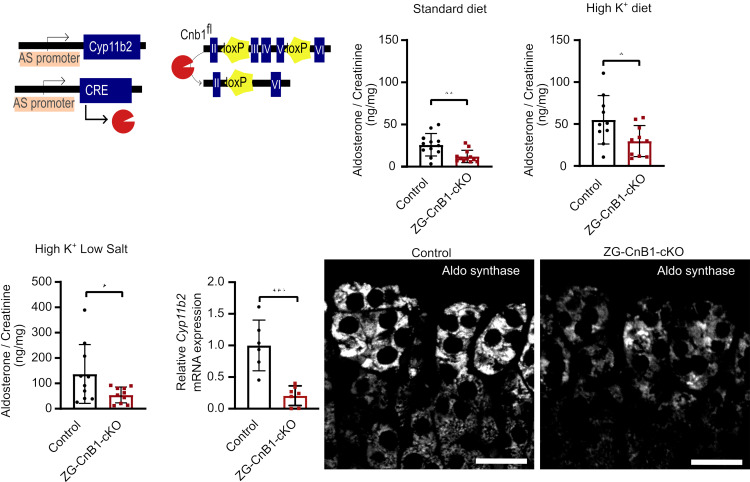
Targeted deletion of CnB1 in mouse ZG impairs the expression of aldosterone synthase and aldosterone production. (**A**) Schematic representation of ZG-CnB1-cKO mouse model. Mice were heterozygous for AS^CreER/+^ and homozygous for Cnb1^fl/fl^ or Cnb1^+/+^ alleles. Constitutive Cre expression and recombination results in excision of exons 3–5 of the Cnb1 gene. A 24-hour urinary aldosterone in female ZG-CnB1-cKO mice (6 to 9 weeks old) under standard diet (*n* = 12) (**B**), HK diet (*n* = 10) (**C**), and HK+LS diet (*n* = 10) (**D**) was assessed via ELISA and adjusted to urinary concentration via normalization to creatinine. Statistical differences assessed by 2-tailed Student’s *t* test. (*P < 0.05; **P < 0.01.) (**E**) Mice were sacrificed under HK+LS diet, and expression of AS in adrenals was assessed by qPCR (*n* = 6–7). Statistical differences assessed by 2-tailed Student’s *t* test. (***P < 0.001.) (**F**) Representative AS immunofluorescence staining in adrenal gland sections from control (*n* = 4) and ZG-CnB1-cKO (*n* = 3) mice. Scale bars, 25 μm. AS, aldosterone synthase.

We first generated a mouse model with a constitutive deletion of CnB1 from ZG (ZG-CnB1-cKO) by crossing animals expressing the Cre recombinase under the control of the *Cyp11b2* promoter (35) with mice carrying *LoxP* sites flanking exons 3 to 5 of *Ppp3r1* (Cnb1^fl/fl^) (34) (Figure 2A). Mice expressing only Cre recombinase were used as control. Female ZG-CnB1-cKO mice secreted less aldosterone in the urine under control conditions, as well as after stimulation of aldosterone production with a K^+^-rich (HK) diet (Figure 2, B and C, respectively). Moreover, aldosterone was the only steroid of adrenal origin affected by the ZG-specific deletion of CnB1, highlighting the specificity of this model (Supplemental Table 2). Female ZG-CnB1-cKO mice were subsequently fed a high-K^+^ + low-salt (as for Na^+^) (HK+LS) diet in order to maximally stimulate the secretion of aldosterone by additional activation of the RAS. Also in this condition, ZG-CnB1-cKO female mice secreted significantly less aldosterone than their control counterparts (Figure 2D). Moreover, the expression of AS was significantly reduced in adrenals of female ZG-CnB1-cKO mice sacrificed on the last day of the HK-LS diet as assessed by qPCR (*Cyp11b2* gene) and immunostaining (Figure 2, E and F, respectively). These results are consistent with the marked decrease in expression of *CYP11B2* observed in NCI-H295R cells treated with tacrolimus for 24 hours (Supplemental Figure 3). Surprisingly, male mice did not exhibit any clinically relevant phenotype (Supplemental Figure 2B).

**Figure 3:**
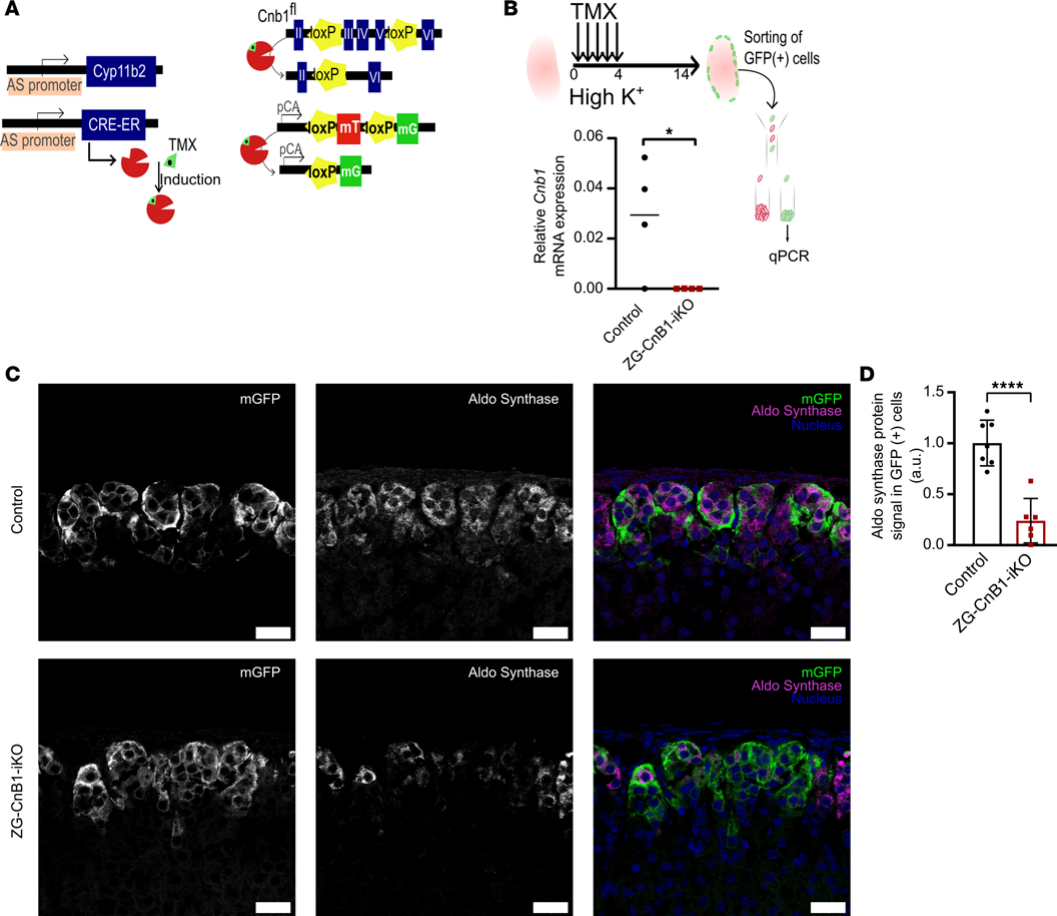
Inducible deletion of CnB1 in mouse ZG impairs the expression of AS. (**A**) Schematic representation of the tamoxifen-inducible ZG-CnB1-iKO mouse model and experimental timeline. Mice were doubly heterozygous for AS^CreER/+^ R26R^mTmG/+^ and homozygous for Cnb1^fl/fl^ or Cnb1^+/+^ alleles. Tamoxifen-induced Cre recombination results in excision of exons 3–5 of the Cnb1 gene, deletion of the mTomato cassette, and expression of the mGFP cassette. TMX, tamoxifen; CRE-ERT, tamoxifen-inducible Cre-estrogen receptor recombinase; mT, membrane-targeted Tomato; mG, membrane-targeted GFP; pCA, chicken promoter core promoter. (**B**) Tamoxifen was administered to female mice (8 to 16 weeks old) fed an HK diet for 5 consecutive days, 2 weeks prior to the experiments. Cells from the adrenal glands of female control and ZG-CnB1-iKO mice on HK diet were isolated (*n* = 4 mice), GFP^+^ cells were sorted, and *Cnb1* mRNA expression was assessed by qPCR. Statistical differences were assessed by 2-tailed Student’s *t* test. (*P < 0.05.) (**C**) Representative AS immunofluorescence staining in adrenal gland sections from control and ZG-Cnb1-KO female mice under HK diet (mGFP signal in green, AS signal in magenta, and nuclear signal in blue). Scale bars, 25 μm. (**D**) Quantification of AS immunofluorescence staining in GFP^+^ ZG cells from control (*n* = 7) and ZG-Cnb1-KO (*n* = 6) mice. Each data point in the graph represents 1 animal. Statistical differences assessed by 2-tailed Student’s *t* test. (****P < 0.0001.)

To rule out an effect of CnB1 deletion during ZG development, we generated ZG-CnB1-iKO (inducible knockout) mice by crossing animals expressing the tamoxifen-inducible CreERT2 enzyme under the control of the *Cyp11b2* promoter (AS^CreER/+^) (contributed by Boston Children’s Hospital) with Cnb1^fl/fl^ mice. ZG-CnB1-iKO mice additionally carried an mTmG lineage reporter allele (R26R^mTmG/+^) (36), allowing for the identification of cells that had undergone recombination by a distinctive GFP membrane fluorescence (Figure 3A). The effective deletion of CnB1 in ZG cells upon tamoxifen induction 6 to 8 weeks after birth was verified by qPCR in sorted GFP^+^ adrenal cells (Figure 3B). Of note, although the recombination efficiency after tamoxifen induction was relatively low (~20% of ZG), the number of recombined ZG cells was comparable between adrenals from control and ZG-CnB1-iKO animals (Supplemental Figure 4A). No major adrenal related changes were found in general physiological parameters between control and ZG-CnB1-iKO mice of either sex, likely due to the low recombination efficiency (Supplemental Tables 3–5).

ZG-CnB1-iKO and control mice were fed an HK diet to stimulate the expression of *Cyp11b2*. Immunofluorescence analysis of control animals showed expression of AS in both recombined (mGFP^+^) and non-recombined (mGFP^–^) ZG cells (Figure 3C). In contrast, AS expression was absent in the majority of GFP^+^ recombined ZG cells in ZG-CnB1-iKO mice (Figure 3C), verified by quantitative immunofluorescence analysis (Figure 3D).

Moreover, the expression of *Cyp11b2* was significantly decreased in adrenals from female ZG-CnB1-iKO mice, as assessed by qPCR (Supplemental Figure 4C). Furthermore, the aldosterone response to stimulation with 5 mM K^+^, ex vivo, was significantly reduced in adrenal glands from female ZG-CnB1-iKO mice compared with control animals (Supplemental Figure 4D).

Taken together, our data indicate that CnB1 deletion hinders the secretion of aldosterone stimulated by K^+^ and RAS activation, in vivo.

### Cn dephosphorylates NFATC4 in response to K^+^ stimulation.

We first studied whether Cn inhibition affects the sensitivity of the plasma membrane to changes in extracellular K^+^. As assessed by whole-cell patch-clamping using the QPatchII automated patch clamp platform, tacrolimus treatment had no effect on the electrophysiological properties of NCI-H295R cells in response to high K^+^ (Supplemental Figure 5, A and B).

**Figure 4:**
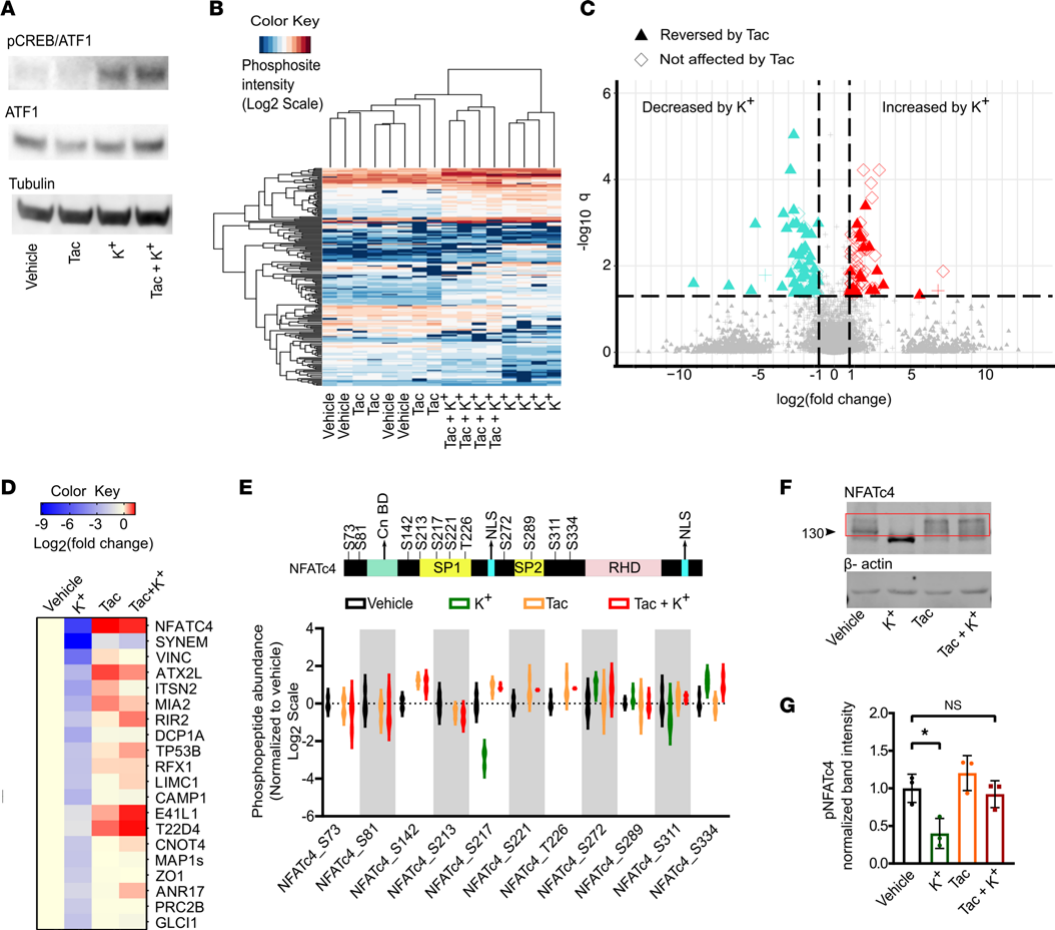
The Cn/NFATC4 pathway is activated upon K^+^ stimulation in NCI-H295R cells. (**A**) Representative immunoblot (*n* = 4) showing phosphorylation of CREB-ATF1 at Ser133 in response to K^+^ stimulation in the presence or absence of tacrolimus in NCI-H295R cells. Tubulin served as a loading control. Molecular weight is indicated on the left in kDa. (**B**) Unsupervised clustering of differentially regulated phosphosites after K^+^ stimulation in the presence or absence of tacrolimus in NCI-H295R cell line (*n* = 4 biological replicates per group). (*q* < 0.05 and log_2_FC > 1.) (**C**) Volcano plot visualization of the phosphosites that have been differentially regulated after K^+^ stimulation. Filled triangles indicate the significantly deregulated phosphosites by K^+^ stimulation and those reversed by tacrolimus treatment while unfilled diamonds indicate the tacrolimus-irreversible deregulated phosphosites after K^+^ stimulation. (**D**) Heatmap of the top 20 proteins dephosphorylated at Ser or Thr after K^+^ stimulation and the dephosphorylation inhibited by tacrolimus treatment. Data are normalized to vehicle. (**E**) Schematic representation of NFATC4 protein domains gathered from UniProt (top). Regulation of all NFATC4 phosphosites found in the phosphoproteomics data for the conditions shown (bottom). P, phosphate; Cn BD, Cn binding domain; SP1 and SP2, serine-proline rich domain; RHD, Rel homology domain; NLS, nuclear localization signal. The phosphorylation of residues S73, S81, S142, S213, S221, and T226 fell below the detection limit of MS upon K^+^ treatment. (**F**) Representative immunoblot showing total NFATC4 and β-actin in response to K^+^ stimulation in the presence or absence of tacrolimus in NCI-H295R cell line. (**G**) Densitometric quantification of phosphorylated NFATC4 bands in red rectangle blot from 3 independent experiments. Statistical differences assessed by 1-way ANOVA with Tukey’s multiple-comparison posttest. (*P < 0.05.)

Next, to study early changes in protein phosphorylation resulting from Cn inhibition, NCI-H295R cells treated with tacrolimus or vehicle were stimulated with 15 mM K^+^ for 30 minutes. We validated previous observations that stimulation of NCI-H295R cells with K^+^ promotes the phosphorylation of CREB/ATF1 (13); however, CREB/ATF1 phosphorylation was not affected by treatment with tacrolimus (Figure 4A). Next, to gain an overview of the changes in protein phosphorylation triggered by high K^+^ stimulation, proteomics and phosphoproteomics approaches were used. No evident changes in protein expression were observed in any of the groups as assessed by quantitative mass spectrometry (Supplemental Data 1). Two-group comparison analysis showed that the phosphorylation of 364 peptides (250 proteins) was significantly changed after K^+^ stimulation (Supplemental Data 2 and Supplemental Figure 6A). Among them, the phosphorylation of 132 peptides was significantly increased while the phosphorylation of 232 phosphopeptides was significantly decreased (Supplemental Figure 6A). Moreover, analysis using the NetworKin bioinformatics tool (37) led to the identification of multiple kinases, in addition to CaMK (16, 17), whose activity was modified by K^+^ stimulation (Supplemental Figure 6B).

Unsupervised hierarchical clustering of significantly regulated phosphopeptides revealed that the phosphoproteome of K^+^-stimulated cells clustered independently from the phosphoproteome of vehicle-treated cells (Figure 4B). In addition, tacrolimus treatment prevented the K^+^-dependent phosphorylation or dephosphorylation of a large subset of phosphopeptides (Figure 4, B and C, and Supplemental Data 3). We then focused on proteins in which K^+^-dependent dephosphorylation was prevented by tacrolimus, aiming to identify direct Cn targets. Within this subset, NFATC4 exhibited the largest difference in Ser/Thr phosphorylation (Figure 4D). In particular, 7 (of a total of 11) Ser/Thr residues were significantly dephosphorylated upon K^+^ treatment. All 7 residues exhibited phosphorylation levels comparable or higher than vehicle conditions (Figure 4E) in cells treated with tacrolimus and K^+^. As previously shown (38), NFATC4 phosphorylation resulted in a diffuse band (smear) on immunoblot with a higher molecular weight than expected for its amino acid sequence, which was lost upon treatment with alkaline phosphatase (Supplemental Figure 7). Treatment of NCI-H295R cells with K^+^ led to a similar effect (Figure 4, F and G), consistent with protein dephosphorylation and in agreement with the phosphoproteomics data (Figure 4, D and E). Furthermore, dephosphorylation of NFATC4 was prevented by incubating NCI-H295R cells with tacrolimus (Figure 4).

Together, our data indicate that Cn dephosphorylates NFATC4 in response to K^+^ treatment.

### NFATC4 is crucial for the stimulation of CYP11B2 expression and aldosterone production.

**Figure 5:**
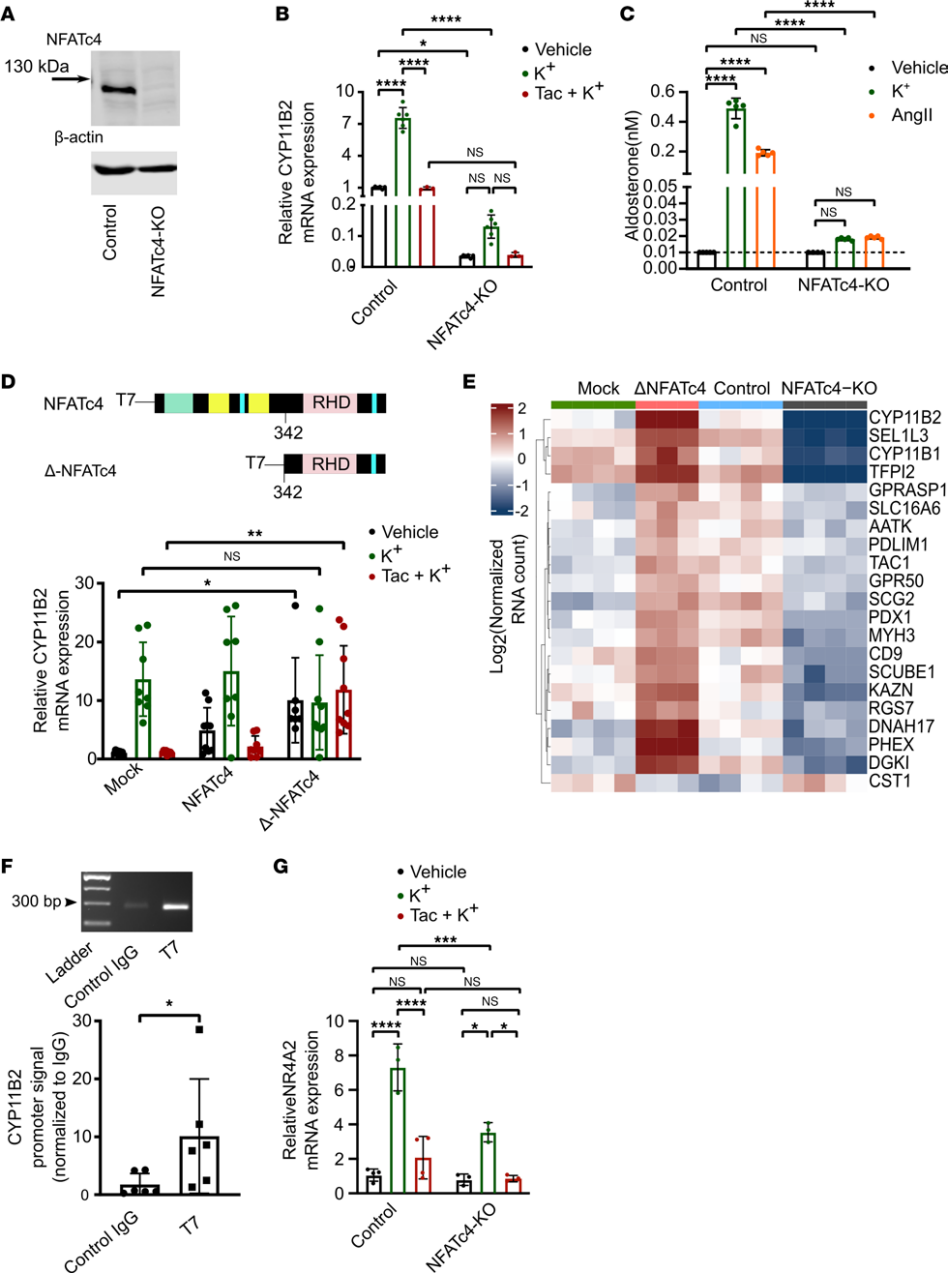
Active NFATC4 is crucial to drive *CYP11B2* expression and aldosterone production in NCI-H295R cells. (**A**) Representative immunoblots (*n* = 3) showing NFATC4 in K^+^-stimulated control and NFATC4-KO NCI-H295R cells. (**B**) *CYP11B2* expression (qPCR) in control and NFATC4-KO NCI-295R cells stimulated with 15 mM K^+^ in the presence or absence of tacrolimus. Statistical differences assessed by 2-way ANOVA with Tukey’s multiple-comparison posttest. (*P < 0.05, ****P < 0.0001; *n* = 3–6.) (**C**) Aldosterone concentration in the supernatant of control and NFATC4-KO NCI-295R cells stimulated with 15 mM K^+^ and 100 nM AngII for 48 hours assessed by ultra-performance liquid chromatography tandem-mass spectrometry (UPLC-MS/MS). The concentration of aldosterone in vehicle-treated cells was below the detection limit. Statistical differences assessed by 2-way ANOVA with Tukey’s multiple-comparison posttest. (****P < 0.0001; *n* = 4–5.) (**D**) *CYP11B2* expression (qPCR) in NCI-H295R cells transiently overexpressing empty (mock), full-length NFATC4, and constitutively active NFATC4 (ΔNFATC4) plasmids, stimulated with 15 mM K^+^ in the presence or absence of tacrolimus (10 nM). Statistical differences assessed by 2-way ANOVA with Tukey’s multiple-comparison posttest. (*P < 0.05, **P < 0.01; *n* = 7–9.) (**E**) Heatmap of the most significantly changed transcripts due to deletion of NFATC4 or overexpression of ΔNFATC4 in NCI-H295R cells as assessed by RNA-Seq. (**F**) Representative gel electrophoresis showing *CYP11B2* promoter enrichment (qPCR) assessed in sheared chromatin of ΔNFATC4-overexpressing cells pulled down with anti-T7 tag antibody or control IgG. In graph, ratio of anti-T7 ab to control IgG. Statistical differences assessed by Mann-Whitney test. (*P < 0.05; *n* = 6.) (**G**) Control and NFATC4-KO NCI-H295R cells were stimulated with 15 mM K^+^ in the presence or absence of tacrolimus (10 nM). *NR4A2* expression levels were determined by qPCR. Statistical differences assessed by 2-way ANOVA with Tukey’s multiple-comparison posttest. (*P < 0.05; ***P < 0.001; ****P < 0.0001; *n* = 3–4.)

To test the role of NFATC4 in the regulation of *CYP11B2* expression and aldosterone production, we used CRISPR/Cas9 technology to inactivate NFATC4 in NCI-H295R cells (Figure 5A). *NFATC4*-knockout (NFATC4-KO) cells, compared with control cells, exhibited a marked decrease in *CYP11B2* expression by qPCR (Figure 5B), which could not be induced by K^+^ stimulation (Figure 5C). In addition, NFATC4-KO cells failed to induce aldosterone production in response to K^+^ and AngII stimulation (Figure 5C). Next, NCI-H295R cells were transfected with either full-length NFATC4 or NFATC4 lacking the N-terminal domain, which renders it constitutively active (Δ-NFATC4) (39). Transfection of NCI-H295R with full-length NFATC4 neither affected the K^+^-stimulated expression of *CYP11B2* nor the effect of tacrolimus (Figure 5D). In contrast, NCI-H295R cells transfected with Δ-NFATC4 exhibited increased expression of *CYP11B2* that was insensitive to either K^+^ stimulation or tacrolimus treatment.

RNA-Seq analysis identified *CYP11B2* as the transcript exhibiting the strongest stimulation in Δ-NFATC4 cells combined with the strongest inhibition in NFATC4-KO cells (Figure 5E), suggesting that NFATC4 directly and specifically modulates the expression of *CYP11B2*. To examine whether NFATC4 directly binds the *CYP11B2* promoter, we first identified putative *NFATC4* binding motifs within the proximal promoter as predicted by the PATCH bioinformatics tool (40). The analysis identified 3 putative *NFATC4* binding motifs between –1,000 and –1,500 bp upstream of the *CYP11B2* transcription start site (Supplemental Figure 9A). NCI-H295R cells were transfected with ΔNFATC4 tagged with the T7 tag sequence (39). qPCR performed after chromatin immunoprecipitation (ChIP) with T7 tag antibody revealed that NFATC4 was enriched in the *CYP11B2* promoter region (Figure 5F). Interestingly, the expression of NR4A2 encoding the Nurr1 transcription factor was modulated in a manner resembling *CYP11B2* expression (Figure 5G). Nurr1 was earlier identified as one of the key regulators of *CYP11B2* expression upon stimulation with AngII (15). As such, our results suggest that NFATC4 regulates the expression of *CYP11B2* in a mixed direct and indirect manner in response to K^+^ stimulation.

Of note, the expression of *CYP11B1* was also markedly decreased in NFATC4-KO cells while it was only slightly, but significantly, increased in ΔNFATC4 cells (Figure 5E and Supplemental Figure 8, A, B, and D). Moreover, the secretion of cortisol in response to 8Br-AMP stimulation was strongly inhibited in NFATC4-KO cells (Supplemental Figure 8E).

## Discussion

**Figure 6:**
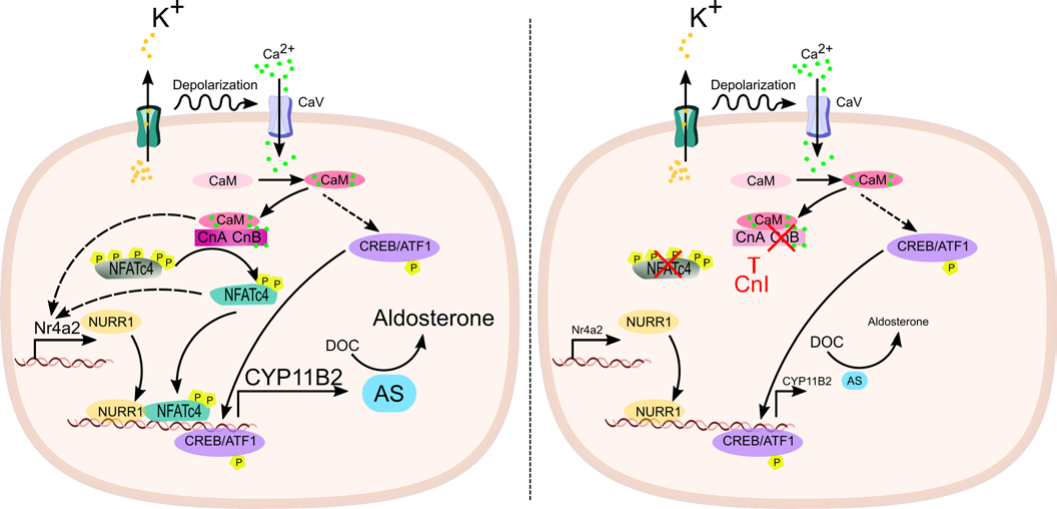
Proposed model of Cn/NFATC4 activation upon K^+^ stimulation in aldosterone-producing adrenal cells. Increased intracellular Ca^2+^ levels upon K^+^ stimulation activates the Cn/NFATC4 pathway to upregulate AS and increases aldosterone production. Inhibition of the Cn/NFATC4 pathway impairs the expression of Nurr1, *CYP11B2*, and AS, ultimately blunting aldosterone biosynthesis.

The present study aimed to decipher the role of Cn in the regulation of aldosterone production. First, we demonstrated that Cn inhibition impairs the stimulation of *CYP11B2* expression and aldosterone production by K^+^ and AngII, in vitro, in NCI-H295R cells. Additionally, tacrolimus inhibited the stimulation of *CYP11B2* expression triggered by high K^+^, ex vivo, in both mouse and human adrenal tissue preparations. Our results are in agreement with a recent finding from Ito and coworkers (31) that reported inhibition of *CYP11B2* expression by tacrolimus in NCI-H295R cells stimulated with K^+^. We now show that cyclosporin A, a chemically and mechanistically distinct inhibitor of Cn (41), also blunts *CYP11B2* expression in a dose-dependent manner. Moreover, using ZG-CnB1-cKO and ZG-CnB1-iKO mouse models, we demonstrate that aldosterone production critically depends on Cn activity, ruling out any off-target effect of CnI. We further identified NFATC4 as an essential signaling factor in the regulation of *CYP11B2* expression. Our data support a mechanism whereby the increase in intracellular Ca^2+^ triggered by plasma membrane depolarization activates Cn, which, in turn, dephosphorylates and activates NFATC4. Activated NFATC4 directly stimulates the expression of *CYP11B2* via binding to its promoter region and indirectly via stimulation of *NR4A2* expression, ultimately promoting aldosterone production (Figure 6).

Despite the overt hypoaldosteronism observed in ZG-CnB1-cKO female mice, they were still able to stimulate aldosterone production under control, HK, and HK+LS diets, though to lower levels than control mice. Moreover, NFATC4-KO cells were still able to stimulate *NR4A2* expression upon K^+^ stimulation. These results speak in favor of alternative mechanisms of stimulation of aldosterone production independent of the Cn/NFATC4 pathway. Indeed, NFATC3 is highly expressed in NCI-H295R cells and could compensate for the lack of NFATC4 (Supplemental Figure 9B), while CREB/ATF1 phosphorylation still occurs in tacrolimus-treated NCI-H295R cells. While other yet-undefined mechanisms might be in play at a systemic level, the hampered production of aldosterone even under maximal stimulation with an HK+LS diet clearly demonstrates that Cn is critical for K^+^ and RAS-stimulated aldosterone production.

Perhaps one of the most striking findings of our study is the marked difference in the stimulation of aldosterone production between male and female mice. In line with our observations, it has been previously reported that adrenal cells acutely dissociated from female rats are significantly more sensitive to AngII and K^+^ than those derived from male animals (42). As such, our data suggest that increased dietary K^+^ intake has a similar effect on aldosterone production in male and female mice but that different pathways mediate it. Whereas in females, an increase in extracellular K^+^ is sufficient to increase aldosterone production, in males, other unidentified factors also play an important role. Interestingly, adrenal adenoma pieces from men were sensitive to ex vivo stimulation with 5 mM K^+^, suggesting a species-related difference. Moreover, tacrolimus blunted this effect, pointing toward a conserved mechanism of regulation of aldosterone production in mice and humans. Unfortunately, our small cohort included only men, precluding the identification of a sex-related pattern in ex vivo stimulation of *CYP11B2* expression. Nevertheless, the underlying molecular mechanisms explaining the differential sensitivity of murine male and female ZG cells to aldosterone secretagogues remains to be elucidated.

In this report, we provide a comprehensive overview of the changes in the phosphorylation landscape in aldosterone-producing cells upon stimulation with K^+^. Our results validated that the targets of CaMKII appeared more phosphorylated upon K^+^ stimulation (5). In contrast, the targets of kinases such as MAPK3, CDK2, CK1, JNK, and GSK3 appeared more dephosphorylated compared with controls. These results suggest that the activity of these kinases is inhibited by K^+^ stimulation. Whether and how these kinases participate in the regulation of aldosterone production is currently not fully understood. We believe that the data sets generated in this study will be of great value for future studies addressing the regulation of steroidogenesis in ZG cells.

We found that the Cn/NFATC4 pathway is critical for the physiological regulation of aldosterone biosynthesis. In agreement with our results, *NFATC4* was found to be upregulated in a mouse model of adrenal hyperplasia expressing constitutively active β-catenin specifically in the ZG (43). Moreover, a study by Briones and coworkers using in vitro–differentiated 3T3-L1 adipocytes suggested that the Cn/NFATC4 pathway may play a role in adipocyte steroidogenesis (44). Taken together, these findings suggest that specific inhibition of the Cn/NFATC4 axis could aid the treatment of diseases featuring inappropriately high secretion of aldosterone. PA is caused by autonomous aldosterone production and is the most frequent cause of secondary hypertension, affecting up to 20% of all patients with treatment-resistant hypertension, representing an enormous global burden. In addition to effects on blood pressure, PA is associated with a higher risk of coronary artery disease, diabetes, and heart failure compared with primary hypertension, underscoring aldosterone’s critical role in these disease processes (45). Nevertheless, the systemic effects of CnI, including their immunosuppressive properties as well as their nephrotoxicity, severely limit their therapeutic use for the treatment of hyperaldosteronism. In recent years, substantial efforts have been made to find novel inhibitors of Cn bypassing the toxic effects of tacrolimus and cyclosporin A. Novel small molecules and peptidyl inhibitors specifically blocking the NFAT/Cn interaction are among the most promising drugs recently developed (46). Since the dysregulation of NFATC4 has been linked to disorders such as breast and ovarian cancer as well as cardiac hypertrophy (47–49), the development of blockers specifically targeting the Cn/NFATC4 interaction could be used as a therapeutic strategy for multiple diseases.

Hyperkalemia is one of the most common adverse effects observed in patients treated with CnI (19). Tacrolimus has been found to cause overactivation of the NaCl cotransporter in the distal convoluted tubule (DCT) of the kidney (50), which is believed to contribute to the onset of hyperkalemia in CnI-treated patients. Moreover, it has been previously shown that CsA and tacrolimus diminish aldosterone-mediated transcriptional activity of the mineralocorticoid receptor (51). Nevertheless, a recent report from our group demonstrated that the targeted inactivation of Cn activity specifically in the DCT is not sufficient to cause hyperkalemia in mice (52). Aldosterone crucially contributes to K^+^ homeostasis by promoting the activity of the epithelial Na^+^ channel and thus Na^+^-driven kaliuresis in the distal nephron (53). Indeed, the most severe cases of PA feature hypokalemia (54) while hypoaldosteronism is associated with hyperkalemia (55). Our current study further supports the hypothesis that the adrenal ZG is a primary target of CnI. The insensitivity to changes in extracellular K^+^ of aldosterone-producing cells upon Cn inhibition likely contributes to the onset of hyperkalemia in patients treated with CnI.

In summary, we found that the Cn/NFATC4 pathway is critical for the regulation of *CYP11B2* expression and aldosterone biosynthesis in ZG cells. Our results may help explain CnI-related hyperkalemia and contribute molecular targets for the treatment of PA.

## Methods

### Cell culture and treatments

The NCI-H295R human adrenocortical carcinoma cell line (ATCC reference: CRL-2128; passage 39–50) was maintained in DMEM-F12 (Gibco) supplemented with 10% FCS (Amimed) and 1× insulin-transferrin-selenium (ITS) (Gibco).

### Plasmids and transfection

The Neon Transfection System (Invitrogen) was used to transfect NCI-H295R cells with the following settings: 2 pulses, 20 milliseconds, and 1,300 V. One million NCI-H295R cells were transfected with 1 μg of plasmid DNA.

For NFATC4 overexpression experiments, 5 × 10^5^ cells were transfected with pcDNA3.T7, pcDNA3.T7 NFAT3 (Addgene plasmid 28224), and pcNDA3.T7 delta-NFAT3 (Addgene plasmid 48138) plasmids and seeded onto 12-well plates. Three days after transfection, the medium was refreshed, and the cells were treated with DMSO (MilliporeSigma, D2650) or tacrolimus for 1 hour before stimulation with 15 mM K^+^ for 6 hours. Afterward, *CYP11B2* expression was determined by qPCR.

The NFATC4-KO NCI-H295R cells were generated using the following guide RNA (gRNA) oligonucleotide sequences, 5′ GGCTGCCTGAGAACAACATGG 3′ and 5′ GATGGCGCTGCACCTATCGGT 3′, by utilizing pSpCas9 (sgRNA) CRISPR plasmid system. Plasmids carrying the 2 gRNAs were cotransfected into NCI-H295R cells to target exons 3 and 5 of NFATC4. Single cells were seeded to a 96-well plate to develop NCI-H295R-NFATC4-KO cell clones. Cells transfected in parallel with empty plasmids served as control.

### Animals

Mice were kept on a 12-hour light/12-hour dark cycle with access to standard chow and tap water.

ZG-CnB1-cKO mice 6–9 weeks old were generated by crossbreeding Cnb1^fl/fl^ mice (34) (The Jackson Laboratory RRID: IMSR_JAX:017692) and ZG-specific AS^Cre+^ mice (35). Control and ZG-CnB1-cKO mice were kept individually in metabolic cages for urinary aldosterone measurements. The 24-hour urine was collected from mice fed with a regular chow diet for 3 days, an HK diet for 4 days, or an HK+LS diet for 3 days. At the end of the experiment, animals were euthanized using CO_2_, and adrenal glands were harvested for qPCR and immunofluorescence analysis.

ZG-CnB1-iKO mice 2–4 months old were generated by crossbreeding Cnb1^fl/fl^ mice (34) (The Jackson Laboratory RRID: IMSR_JAX:017692) and ZG-specific tamoxifen-inducible AS^CreER/+^ mice (contributed by Boston Children’s Hospital) and R26R^mTmG/+^ mice to generate triple-transgenic AS^CreER/+^ R26R^mTmG/+^ Cnb1^fl/fl^ mice. AS^CreER/+^ R26R^mTmG/+^ Cnb1^+/+^ mice were used as controls. AS^CreER/+^ mice were maintained in a heterozygous state in all experiments. We administered 2 mg of tamoxifen (Thermo Fisher Scientific) in EtOH/sunflower oil (1:10) to mice fed an HK diet via gastric gavage for 5 days, 2 weeks prior to the experiment.

### Diets

Potassium-deficient diet (K^+^ content < 0.02%) was purchased at Ssniff Spezialdiaeten.

High-K^+^ diet (K^+^ 4%, catalog 1818435-203) and low-Na^+^ diet (Na^+^ 0.07%, catalog 1817204-203) were purchased from TestDiet. HK+LS diet was prepared by mincing 100 g of LS diet (K^+^ content 0.91%) mixed with 100 g of 5.9% KCl in water (w/w) to obtain a dry K^+^ content of 4%.

HK diet for ZG-CnB1-iKO animals was prepared by mincing 100 g of standard chow (K^+^ content 0.78%) mixed with 100 g of 4.2% KCl in water (w/w) to obtain a dry K^+^ content of 3%.

### Cell sorting

Adrenal glands of mTmG^+^ animals were dissected under anesthesia and cut into 4 pieces in cold PBS. Adrenal pieces were transferred into DMEM-F12 containing 2 mg/mL collagenase (MilliporeSigma) and shaken at 37°C for 30 minutes. Then, the tissue was further dissociated by pipetting with a 1 mL micropipette tip. DMEM-F12 containing 10% FCS was added to stop the collagenase activity, and single cells were collected via centrifugation at 500*g* for 5 minutes at 4°C after passing the suspension through a 100-micron cell strainer. Afterward, cells were resuspended in PBS containing 1% FCS. GFP^+^ cells were sorted with FACSAria III 5L (BD Biosciences) directly into RNA lysis buffer for further analysis. Animals not induced with tamoxifen were used as negative controls.

### Aldosterone ELISA

The adrenal glands from tamoxifen-induced 2- to 6-month-old control and ZG-CnB1-iKO animals were dissected under anesthesia. Afterward, adrenal glands were incubated ex vivo as described earlier with modifications (56). Briefly, the surrounding fat tissue was removed, and each adrenal gland was cut into 4 pieces in ice-cold oxygenated RING-like buffer (98.5 mM NaCl, 35 mM NaHCO_3_, 3 mM KCl, 1 mM NaH_2_PO4, 2.5 mM CaCl_2_, 1.8 mM MgCl_2_, 25 mM glucose). Adrenal pieces were incubated with a buffer containing 50 mL DMEM, 50 mL Krebs-HEPES solution (95 mM NaCl, 1.8 mM CaCl_2_, 25 mM NaHCO_3_, 1 mM Na_2_HPO_4_, 20 mM HEPES, 0.8 mM MgSO_4_, 0.7 mM KCl), 150 mg d-glucose, and 5% FCS and 1× ITS for 45 minutes at 37°C with 5% CO_2_. A total of 150 μL supernatant was sampled to determine the baseline aldosterone production. Afterward, the adrenal pieces were stimulated with 5 mM K^+^ for 135 minutes, and the supernatants were collected for aldosterone measurements. Supernatants of the cells and adrenal glands were diluted 1:5, and aldosterone levels were measured by aldosterone ELISA kit (Cayman Chemical).

Urine samples of control and ZG-CnB1-cKO mice were treated with HCl overnight to convert the aldosterone-18-glucuronide into free aldosterone and neutralized by NaOH. Afterward, the samples were diluted 1:50 or 1:100 in ELISA buffer, and aldosterone levels were measured by aldosterone ELISA kit (Cayman Chemical). Aldosterone levels were normalized to urinary creatinine measured by calorimetric assay kit (Cayman Chemical).

### Steroid profiling

Control and NFATC4-KO NCI-H295R cells were subcultured onto 12-well plates at a density of 5 × 10^5^. One day later, cells were stimulated with KCl (15 mM) or the same amount of NaCl (mock) for 48 hours. Supernatant of the cells was collected for aldosterone measurement.

Steroids were quantified as described earlier with minor modifications (57). Samples from serum (100 μL) or cell culture (500 μL) were mixed with protein precipitation solution (100 μL, 0.8 M zinc sulfate in water/methanol; 50/50, v/v) supplemented with deuterium-labeled internal standard (ISTD). Urinary samples (200 μL) were diluted with sodium acetate (1,300 μL, 0.1 mM, pH 4.3) and underwent enzymatic digestion with β-glucuronidase from Helix promatia (80 μL; ≥100,000 U/mL, 2 hours, at 55°C, at 850 rpm). Urinary samples were subsequently spiked with ISTD in sodium acetate buffer. Prior to solid phase extraction (SPE) cell culture and serum samples were diluted with water and urinary samples with sodium acetate buffer to a final volume of 1 mL or 2 mL, respectively. Prior to SPE all samples were incubated in a thermoshaker (4°C, 10 minutes, 1,350 rpm) and centrifuged (18,000*g*, 10 minutes, 4°C). Supernatants were transferred to Oasis HBL SPE (3 cc) cartridges (Waters). Columns for serum and cell culture samples were washed with water and methanol/water; SPE columns of urinary samples were washed with water, water containing 2% ammonium hydroxide, and water/methanol/ammonium hydroxide (20% v/v/v) followed by elution (2× 750 μL) of plasma and cell culture steroids using methanol and ethyl acetate for urinary steroids. After evaporation of solvent and reconstitution of steroids in methanol, steroid content was analyzed by UPLC-MS/MS on an Agilent 1290 Infinity II UPLC coupled to an Agilent 6495 triple-quadrupole mass spectrometer equipped with a jet stream electrospray ionization interface (Agilent Technologies). Analyte separation was achieved using a reverse-phase column (1.7 μm, 2.1 mm × 150 mm; Acquity UPLC BEH C18; Waters). Data acquisition and quantitative analysis were performed by MassHunter (Version B.10.0, Build 10.0.27, Agilent Technologies). The lower limit of quantification divided by half (0.01 nM) was used as nominal value for statistical analysis.

### Immunofluorescence assay

Adrenal glands of control and ZG-CnB1-cKO animals were harvested and placed into cold PBS. After trimming the fat tissue around the glands, adrenals were cut into halves with a blade and fixed in 4% paraformaldehyde (PFA) overnight at 4°C. Paraffin section immunofluorescence was performed as described previously (58) with the following modification: antigen retrieval was performed with 10 mM diethanolamine and 1 mM EDTA buffer (pH 9.0). We used 1/100 diluted AS rabbit antibody (gift from Celso Gomez-Sanchez, University of Mississippi Medical Center, Jackson, Mississippi, USA) and Alexa Fluor 647–conjugated goat anti-rabbit IgG (Invitrogen catalog A-21443) were used as primary and secondary antibodies, respectively.

After incision of the inferior vena cava, anesthetized control and ZG-CnB1-iKO mice were sacrificed by exsanguination by perfusion with 20 mL of PBS. Then, mice were perfused with 30 mL of 3% PFA in PBS. Five minutes after perfusion, PFA was rinsed with 20 mL of PBS. All the solutions included 10 IU/mL of heparin (HEPARIN Bichsel 1,000 IE/mL sterile, Grosse Apotheke G. Bichsel AG) and were administered through a polyethylene catheter inserted into the abdominal aorta, at a constant flow rate of 10 mL/min assured with a roller pump (Ismatec SA). Adrenal glands of the animals were harvested and placed in ice-cold PBS. Then, the glands were snap-frozen in liquid isobutane with mounting medium (VWR Chemicals) for cryosectioning.

Adrenal glands were sliced into 6 μm–thick sections using the cryostat microtome (Thermo Fisher Scientific), and sections were placed in ice-cold PBS. Tissue sections were permeabilized with PBS including 0.1% Tween (PBS-T) detergent for 5 minutes. Then, tissues were incubated with the 1/100 diluted AS rabbit antibody (gift from Celso Gomez-Sanchez, University of Mississippi Medical Center, Jackson, Mississippi, USA) in 1% BSA (w/v) in PBS-T overnight at 4°C. Sections were washed with PBS 3 times and incubated with 1/200 diluted anti-rabbit Alexa Fluor 647 antibody (Jackson ImmunoResearch, 711-611-152) and 20 μM Hoechst 33342 (Thermo Fisher Scientific H3570). Then, coverslips were mounted onto the slides with glycergel mounting medium (Dako, C0563) following 3 PBS washes. Antibodies used in this study are listed in Supplemental Table 6.

### Image acquisition and quantification

Imaging for the AS signal in adrenal glands presented in Figure 2 (ZG-CnB1-cKO) was performed using an LSM 510 confocal microscope (Carl Zeiss AG) equipped with 40×/1.3 oil immersion PLAN-APOCHROMAT objective.

To quantify the AS signal presented in Figure 3 (ZG-CnB1-iKO mice), imaging was performed using a DMi8 CS SP8 inverted confocal microscope (Leica Microsystems) equipped with a 63× HC PL APO corr CS2 oil immersion objective. Regions of interest (ROIs) were manually placed on at least 50 GFP^+^ ZG cells per animal. Mean AS fluorescence intensity of the ROIs was determined, and background signal was subtracted from the specific signal. Data were normalized to the mean AS fluorescence signal of control animals.

### Mouse adrenal gland and human patient adrenal tumor ex vivo perifusion

Eight-week-old female C57BL/6 mice were fed with K^+^-deficient food overnight, and the adrenal glands were dissected under anesthesia. After dissection, surrounding fat tissue was removed, and each adrenal gland was cut into 4 pieces in ice-cold adrenal gland perifusion buffer (AGPB) containing 46.9 mL MEM, 53.1 mL Krebs-HEPES solution (95 mM NaCl, 1.8 mM CaCl_2_, 25 mM NaHCO_3_, 1 mM Na_2_HPO_4_, 20 mM HEPES, 0.8 mM MgSO_4_, 0.7 mM KCl), 150 mg d-glucose, and 10 mg BSA.

Adrenal fragments were incubated in glass columns filled with Sephadex G25 fine beads (MilliporeSigma) at 37°C. AGPB was bubbled with medical carbogen (5% CO_2_ and 95% O_2_) and applied at a constant flow rate of 0.15 mL/min. The experimental protocol included 30-minute perifusion with AGPB, then 60-minute perifusion with medium containing tacrolimus or vehicle, followed by stimulation with 5 mM K^+^, 5 mM K^+^, and tacrolimus or vehicle (3 mM K^+^) for 90 minutes.

Freshly resected adrenal tumors were cut into 3 pieces in ice-cold AGPB. Tumor fragments were incubated in glass columns filled with Sephadex G25 fine beads (MilliporeSigma) at 37°C. AGPB was bubbled with medical carbogen (5% CO_2_ and 95% O_2_) and applied at a constant flow rate of 0.15 mL/min. The experimental protocol included 30 minutes perifusion with AGPB, then 1 hour perifusion with medium containing tacrolimus or vehicle, followed by stimulation with 5 mM K^+^ or vehicle (3 mM K^+^).

### RNA isolation and qPCR

Whole adrenal glands from control and ZG-CnB1-cKO mice were directly homogenized in TRI reagent (MilliporeSigma) with steel beads (QIAGEN) with tissue homogenizer (Tissue Lyser II, QIAGEN).

Whole adrenal glands and half of the kidneys from control and ZG-CnB1-iKO mice were directly homogenized in RNA lysis buffer including 1:100 (v/v) β-mercaptoethanol with ceramic beads (Omni International) with tissue homogenizer (Precellys 24, Bertin Instruments) (2 × 20 seconds, 2,000*g*) and centrifuged at 16,000*g*.

For the protein phosphatase screening assay, cells were subcultured onto 6-well plates at a density of 5 × 10^5^. Three days later, cells were treated with 10 nM okadaic acid (Enzo Life Sciences), 5 μM BCI (MilliporeSigma), 1 nM calyculin A (Enzo Life Sciences), 10 nM tacrolimus (Selleckchem), 10 μM sodium orthovanadate (MilliporeSigma), 10 nM fostriecin (Tocris), 50 nM cyclosporine A (Tocris), or vehicle for 1 hour. Afterward, cells were stimulated with 15 mM K^+^ for 6 hours in the presence of the PPi.

To determine dose-dependent effects of CnI, cells were treated with 1, 10, and 100 nM tacrolimus; 5, 50, and 500 nM cyclosporine A; or vehicle for 1 hour. Afterward, cells were stimulated with 15 mM K^+^ for 6 hours in the presence of the CnI.

Total RNA was isolated from the homogenized tissues and from the cell culture samples using NucleoSpin RNA isolation kit (Machery-Nagel) or a Direct-zol RNA miniprep kit (Zymo Research), following the manufacturer’s protocol. A total of 300 ng of RNA was reverse-transcribed into cDNA using GoScript Reverse Transcriptase Kit (Promega) or the High-Capacity cDNA Reverse Transcription Kit (Applied Biosystems). Gene expression was assessed by quantitative real-time PCR using SYBR Green I Master (Roche) and Lightcycler 96 (Roche) or TaqMan Universal PCR Master Mix (Applied Biosystems) and QuantStudio 6 Flex thermocycler (Applied Biosystems). *GAPDH*, *Gapdh*, or *Actb* was used as a housekeeping gene, and data were expressed using either 2^-ΔΔCt^ or 2^-ΔCt^ method (59). Water instead of cDNA was used as a negative control for qPCR experiments. Primers and TaqMan probes used in this study are listed in Supplemental Tables 7 and 8, respectively.

### Alkaline phosphatase treatment

NCI-H295R cells were collected from a 100 mm cell culture plate with RIPA buffer by scraping and homogenized with a 28G syringe on ice. A total of 20 μg protein containing cell extract was treated with calf intestine alkaline phosphatase for 1 hour at 37°C to dephosphorylate the proteins.

### Western blot

For Western blot experiments, cells were starved for 16 hours with DMEM-F12 including 1× ITS before specific treatments. Then, tacrolimus or DMSO was added to the medium of the cells 1 hours before the stimulation with K^+^ (15 mM) for 30 minutes.

Cells were collected by scraping from 6-well plates with SDS lysis buffer (4% [w/v] SDS, 100 mM Tris/HCL pH 8.2, 0.1 M DTT) supplemented with protease inhibitor cocktail (Roche, 10618200) and phosphatase inhibitor (Thermo Fisher Scientific). Cell lysate was homogenized with a 28G syringe on ice. Lysates were boiled at 95°C for 10 minutes, subjected to SDS-PAGE, and transferred to a nitrocellulose membrane. The membranes were blocked with Intercept Protein Free Blocking Buffer (LI-COR Biosciences) diluted with PBS (1:1) for 15 minutes and incubated with antibodies against p-CREB (Cell Signaling Technology), NFATC4 (Abcam) and β-actin (MilliporeSigma) (Supplemental Table 6) overnight at 4°C. Then, membranes were incubated with conjugated secondary antibodies (goat-anti-rabbit IRDye 800 and goat-anti-mouse IRDye 680, both from LI-COR Biosciences) in casein blocking solution for 2 hours at room temperature. After washing 3 times with PBS-T (1:1,000), membranes were scanned with Odyssey CLx imaging system (LI-COR Biosciences), and densitometry of the bands was analyzed with ImageJ (NIH). See complete unedited blots in the supplemental material.

Phosphoproteomics analysis

#### Treatments.

NCI-H295R cells were starved with DMEM-F12 medium including 1% ITS for 16 hours before 1-hour incubation with 10 nM tacrolimus or DMSO. Then, cells were stimulated with KCl (15 mM: final conc.), or the same amount of NaCl was added to medium of nonstimulated cells to keep medium osmolarity constant. At 30 minutes after the stimulation cells were collected with 450 μL SDS lysis buffer. Cells were boiled at 95°C for 10 minutes and kept at –80°C.

#### Protein extraction, digestion, and phosphopeptide enrichment.

For each sample, 500 μg of proteins were used for on-filter digestion using an adaptation of the filter-aided sample preparation protocol (60). Digestion was performed overnight in a wet chamber at room temperature, and peptides were eluted by centrifugation at 14,000*g* for 20 minutes. After elution, 5 μL of peptide mixture was taken and stored for later MS analysis of the proteome. The remaining volume was dried almost to completeness for enrichment of the phosphopeptides. The phosphopeptide enrichment was performed using a KingFisher Flex System (Thermo Fisher Scientific) and MagReSyn Ti-IMAC beads (ReSyn Biosciences) (61).

#### Liquid chromatography-mass spectrometry analysis.

MS analysis of the phosphoproteomics and proteome samples was performed on an Orbitrap Fusion Lumos (Thermo Fisher Scientific) equipped with a Digital PicoView source (New Objective) and coupled to an M-Class UPLC (Waters). The MS proteomics data were handled using the local laboratory information management system (62).

#### Protein and phosphopeptide identification and label-free quantification.

The acquired raw MS data were processed by MaxQuant (version 1.6.2.3), followed by protein identification using the integrated Andromeda search engine (63). Spectra were searched against a canonical UniProt reference proteome of *Homo sapiens* (UP000005640, version 2016-12-09), concatenated to common protein contaminants. The maximum FDR was set to 0.01 for peptides and 0.05 for proteins. Label-free quantification was enabled, and a 2-minute window for match between runs was applied.

#### Data analysis.

In the MaxQuant experimental design template, each file is kept separate in the experimental design to obtain individual quantitative values. Protein fold-changes were computed based on intensity values reported in the proteinGroups.txt file. A set of functions implemented in the R package SRMService (64) was used to filter for proteins with 2 or more peptides allowing for a maximum of 4 missing values, normalizing the data with a modified robust *z* score transformation and computing *P* values using the moderated *t* test with pooled variance (as implemented in the limma package, ref. 65). For the phosphosite analysis a similar data analysis strategy, as described previously (66), was implemented. In brief, the MaxQuant phospho_STY_site.txt file was used as the input file. The phosphosite table was expanded with respect to multiplicity and filtered for a minimum localization site probability of 0.75. For each 2-group comparison all peptides with a maximum of 4 missing values were retained. The data (like for the total proteome) were normalized with a modified robust *z* score transformation, and *P* values were computed with a moderated *t* test with pooled variance (as implemented in the limma package, ref. 65). Calculated *P* values were adjusted for multiple testing using the Benjamini-Hochberg method.

Cutoff values for significantly regulated proteins and phosphopeptides were chosen as fold-change > 2 and *q* < 0.05. Further analysis was done based on that information. Hierarchical clustering and heatmaps were generated using heatmap.2 function in gplots package (67). The volcano plot was created with the EnhancedVolcano package (68).

#### NetworKin analysis.

The significantly upregulated and downregulated phosphopeptides after K^+^ stimulation were separately searched against human ENSEMBL 74 database with the NetworKin high-throughput workflow (37). The possible kinases responsible for the phosphorylation of these residues were selected with a NetworKin score > 1 criterion.

### ChIP-qPCR experiment

A total of 10 × 10^6^ NCI-H295R cells were transfected with 10 μg of pcNDA3.T7 delta-NFAT3 plasmids and seeded onto a 150 mm cell culture dish. At 4 days after the transfection, cells were starved with DMEM-F12 including ITS for 16 hours. Afterward, a ChIP assay was performed using a modified fast-ChIP method (69). Briefly, cells were fixed with 1% formaldehyde for 15 minutes, and formaldehyde was quenched with 125 mM glycine (Biosolve). Cells were washed 3 times with cold PBS and collected in ice-cold PBS by scraping. After centrifugation (2,000*g* for 5 minutes at 4°C), cells were lysed with 1 mL IP buffer containing 50 mM Tris-HCl (pH 7.5), 150 mM NaCl, NP-40 (0.5% v/v), 5 mM EDTA, Triton X-100 (1.0% v/v). Then nuclear pellet was collected with centrifugation (12,000*g* for 1 minute at 4°C) and washed with 1 mL IP buffer by resuspending the pellet and follow-up centrifugation. Chromatin was sheared with 11 rounds of sonication (15-second pulses and 2-minute breaks on ice) each at 60% pulses 90% duty cycle with Sonopuls HD 2070 ultrasonic homogenizer (Bandelin). Lysate was cleared by centrifugation (12,000*g* for 10 minutes at 4°C). A total of 400 μL of the supernatant was incubated with 10 μg anti-T7 (Abcam, ab9138) antibody or with 10 μg goat IgG isotype control (R&D Systems, AB-108-C) for 30 minutes in ultrasonic water bath and 4 hours on the rotating platform at 4°C. Protein G agarose beads (Agarose Bead Technologies) were washed with IP buffer 3 times, and 50 μL beads was added into the antibody-chromatin mixture. After overnight incubation of the beads with the samples on the rotating platform at 4°C, beads were washed with cold IP buffer 6 times. A total of 100 μL 10% (w/v) Chelex 100 slurry (MilliporeSigma) was added onto the washed beads, and they were boiled at 95°C for 10 minutes after 10-second vortex. Afterward, samples were treated with proteinase K (Roche) for 30 minutes at 55°C and afterward boiled for 15 minutes to inactivate the enzyme. Samples were centrifuged at 12,000*g* for 1 minute at 4°C, and 80 μL supernatant was transferred into clean tubes. The remaining DNA on the beads was collected with 120 μL nuclease-free water, vortexing, and centrifugation. Samples were pooled and kept at –20°C.

Putative NFATC4 binding motifs in the *CYP11B2* promoter region were predicted using the transcription factor database, TRANSFAC (PATCH program) (40). Quantification of the predicted regions were determined with *CYP11B2* promoter primers using SYBR Green (Roche) with a real-time PCR machine (LightCycler 96, Roche). Primers used in this study are listed in Supplemental Table 7.

### RNA-Seq

RNA-Seq was performed by the Functional Genomics Center Zurich. RNA was extracted using the QIAGEN RNeasy Mini Kit following manufacturer’s protocol. Extracted RNA was prepared for sequencing using the Illumina TruSeq Stranded mRNA Library Prep assay following manufacturer’s protocol. Sequencing was performed on the Illumina NovaSeq 6000 using the S1 Reagent Kit v1.5 (100 cycles) as per manufacturer’s protocol. Demultiplexing was performed using the Illumina bcl2fastq Conversion Software. Individual library size ranged from 21.2 million to 31.3 million reads.

RNA-Seq analysis was performed using the SUSHI framework (70), which encompassed the following steps: read quality inspected using FastQC and sequencing adaptors removed using fastp; alignment of the RNA-Seq reads using the STAR aligner (71) and with the GENCODE human genome build GRCh38.p13 (Release 37) as the reference (72); counting of gene-level expression values using the featureCounts function of the R package Rsubread (73); and differential expression using the generalized linear model as implemented by the DESeq2 Bioconductor R package (74).

Cutoff values for significantly regulated genes were fold-change > 2 and FDR value < 0.05.

### Statistics

No power analyses were performed. The statistical analysis for the phosphoproteomics analysis is explained in the relevant Methods subsection. Prism 9 software (GraphPad) was used for all other statistical analyses. All data are included without any exclusion method. Details of statistical analyses can be found in each figure legend, and *n* values represent the number of independent, mice, samples, or experiments. Data are presented as mean ± SD unless otherwise specified.

### Study approval

Animal experiments were conducted according to Swiss Laws and approved by the veterinary administration of the Canton of Zurich, Switzerland (license number ZH091/18). All animal procedures were approved by Boston Children’s Hospital’s Institutional Animal Care and Use Committee. The studies done with human adrenal samples were approved by the ethics commission of the Canton of Zurich, Switzerland (BASEC-Nr. 2017-00771).

After obtaining written informed consent, we collected adrenal tissues from patients undergoing adrenalectomy for aldosterone-producing adenomas at the University Hospital Zurich. The diagnosis of PA/aldosterone-producing adenomas was established in accordance with institutional and international guidelines.

### Data availability

Proteomics and phosphoproteomics data have been deposited to the ProteomeXchange Consortium via the PRIDE partner repository with the data set identifier PXD027856. RNA-Seq data are available in the NCBI’s Genome Expression Omnibus repository with the accession number GSE225735. Values for all data points in graphs can be found in the Supporting Data Values file.

## Author contributions

DP conceived the study; MB, FB, JL, and DP designed research; MB, AW, and DP performed research, SL, DVW, AO, and DTB contributed new analytic tools, MB and DP analyzed data; MB, DTB, and DP wrote the manuscript; and all authors reviewed the manuscript.

## Supplementary Material

Supplemental data

Supplemental data 1

Supplemental data 2

Supplemental data 3

Supporting data values

## Version 1 06/13/2023

In-Press Preview

## Version 2 07/24/2023

Electronic publication
