# Supplementary material for: Calcineurin regulates aldosterone production via dephosphorylation of NFATC4
Source: JCI Insight. 2023 Jul 24;8(14):e157027. doi: 10.1172/jci.insight.157027 (PMC10443813; doi:10.1172/jci.insight.157027)

Supplementary information

Manuscript Title: Calcineurin regulates aldosterone production via dephosphorylation of NFATc4

Mesut Berber[1,2,8], Sining Leng[3], Agnieszka Wengi[1], Denise V. Winter[4], Alex Odermatt[4], Felix Beuschlein[2,5,6], Johannes Loffing[1,2], David T. Breault[3,7,*], David Penton[1,2,9,*]

[1]Institute of Anatomy, University of Zurich, Switzerland,

[2]Swiss National Centre for Competence in Research "Kidney Control of Homeostasis" (NCCR Kidney.CH)

[3]Department of Pediatrics, Harvard Medical School and Division of Endocrinology, Boston Children's Hospital, Boston, Massachusetts, USA

[4]Swiss Centre for Applied Human Toxicology and Division of Molecular and Systems Toxicology, Department of Pharmaceutical Sciences, University of Basel, Basel, Switzerland

[5]Department of Endocrinology, Diabetology and Clinical Nutrition, University Hospital Zurich (USZ) and University of Zurich (UZH), Zurich, Switzerland

[6]Medizinische Klinik und Poliklinik IV, Klinikum der Universität München, Munich, Germany

[7]Harvard Stem Cell Institute, Cambridge, Massachusetts, USA

[8]Current address: Department of Pediatrics, Harvard Medical School and Division of Endocrinology, Boston Children's Hospital, Boston, Massachusetts, USA.

[9]Current address: Electrophysiology Facility (e-phac), University of Zurich, Switzerland

*David T. Breault and David Penton contributed equally to this study

To whom correspondence should be addressed:

Dr. David Penton

Electrophysiology Facility

University of Zurich

Winterthurerstrasse 190

8057 Zurich

Email: david.pentonribas@uzh.ch

Phone : +41 44 63 54835

Contents

Supplementary Figure 1: Tacrolimus blunts K+ and AngII-stimulated CYP11B2 upregulation in NCI-H295R cell line ... 3

Supplementary Figure 2: Characterization of ZG-CnB1-cKO animals ... 4

Supplementary Figure 3: 24h tacrolimus treatment blunts baseline CYP11B2 expression in NCI-H295R cells. ... 5

Supplementary Figure 4: Characterization of ZG-CnB1-iKO mice ... 6

Supplementary Figure 5: Tacrolimus treatment does not affect cell membrane sensitivity to changes in extracellular K+ concentrations ... 7

Supplementary Figure 6: Two group comparison of phosphoproteomic data and kinome analysis following K+ stimulation ... 8

Supplementary Figure 7: Immunoblot profile of NFATc4 is changed upon treatment with alkaline phosphatase ... 9

Supplementary Figure 8: Role of NFATc4 in steroidogenesis of NCI-H295R cells ... 10

Supplementary Figure 9: NFATc4 binding motifs in CYP11B2 promoter and expression of NFAT isoforms in NCI-H295R cells ... 11

Supplementary Table 1: Patient information of adrenal tissues used ex vivo incubation ... 12

Supplementary Table 2. Urinary steroid profile of female ZG-CnB1-cKO mice under standard and high K+ diet measured by UPLC-MS/MS ... 13

Supplementary Table 3: Blood parameters of ZG-CnB1-cKO mice under standard diet All values are given as mean ± SD.) ... 14

Supplementary Table 4: Blood Physiological parameters of ZG-CnB1-iKO mice under high K+ diet ... 15

Supplementary Table 5. Plasma steroid profile of ZG-CnB1-iKO mice under high K+ diet measured by UPLC-MS/MS ... 16

Supplementary Table 6. Antibody list ... 17

Supplementary Table 7. Primer list ... 18

Supplementary Table 8. TaqMan gene expression assay list (Applied biosystems) ... 19

Supplementary Methods ... 20

- Automated Patch clamp ... 20
- Generation of AS-CreER mouse model ... 20

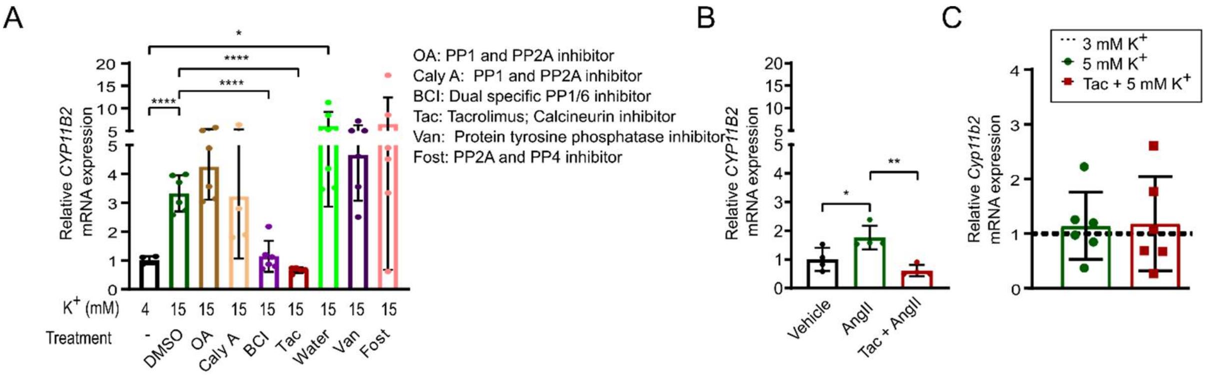

Supplementary Figure 1: Tacrolimus blunts K$^+$ and AngII-stimulated CYP11B2 upregulation in NCI-H295R cell line. **(A)** NCI-H295R cells were stimulated with 15 mM K$^+$ in the presence of okadaic acid (OA), calyculin A (Caly A), E)-2-Benzylidene-3-(cyclohexylamino)-2,3-dihydro-1H-inden-1-one (BCI), tacrolimus (Tac), sodium orthovanadate (Van) and fostriecin (Fost) for 6 h after preincubation with these protein phosphatase inhibitors for 1h (n=3-6). DMSO and water were used as vehicle control. *CYP11B2* expression levels were determined by qPCR. Statistical differences assessed by Student`s t-test. (*p<0.05, ****p < 0.0001) **(B)** NCI-H295R cells were pre-incubated with vehicle or 10 nM tacrolimus for 1h before stimulation with 10 nM AngII for 6h in the presence of vehicle or tacrolimus respectively. . Statistical differences assessed by one-way ANOVA with Tukey`s multiple comparison post-test (n=4, *p<0.05, **p<0.01) **(C)** Adrenal glands fragments from male C57BL/6 mice were perifused, *ex vivo,* with buffer containing vehicle or Tacrolimus (10 nM) for 60 min followed by buffer containing 5 mM K$^+$ or 5 mM K$^+$ + Tac for 90 min (n=6). The contralateral adrenal from each mouse was perifused with 3 mM K$^+$ as a control. *Cyp11b2* expression levels were determined by qPCR.

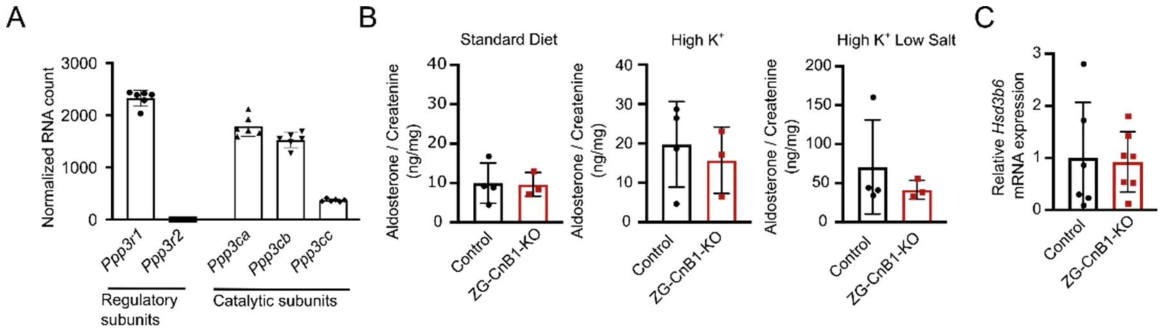

Supplementary Figure 2: Characterization of ZG-CnB1-cKO animals **(A)** Expression pattern of regulatory and catalytic subunits of calcineurin in female mouse adrenal glands. (Reanalysis of RNAseq Data: GSE144503 (Leng S. et al., 2020)). **(B)** 24h urinary aldosterone in male ZG-CnB1-cKO mice under standard diet, high K^+ diet and HK+LS diet was assessed via ELISA and adjusted to urinary concentration via normalization to creatinine. Statistical differences assessed by Student`s t-test. **(C)** Female mice were sacrificed under HK+LS diet and expression of hydroxy-delta-5-steroid dehydrogenase, 3 beta- and steroid delta-isomerase 6 (Hsd3b6) in adrenals was assessed by quantitative PCR**.**

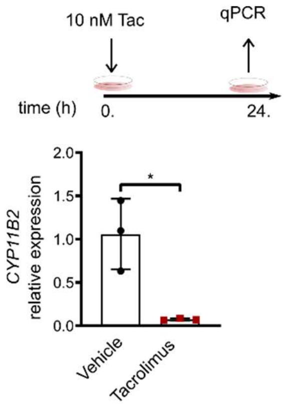

Supplementary Figure 3: 24h tacrolimus treatment blunts baseline CYP11B2 expression in NCI-H295R cells. NCI-H295R cells were treated with 10 nM tacrolimus or vehicle (DMSO) for 24 h. CYP11B2 mRNA expression levels were determined by qPCR. Statistical differences assessed by Student`s t-test. (n=3, *p<0.05)

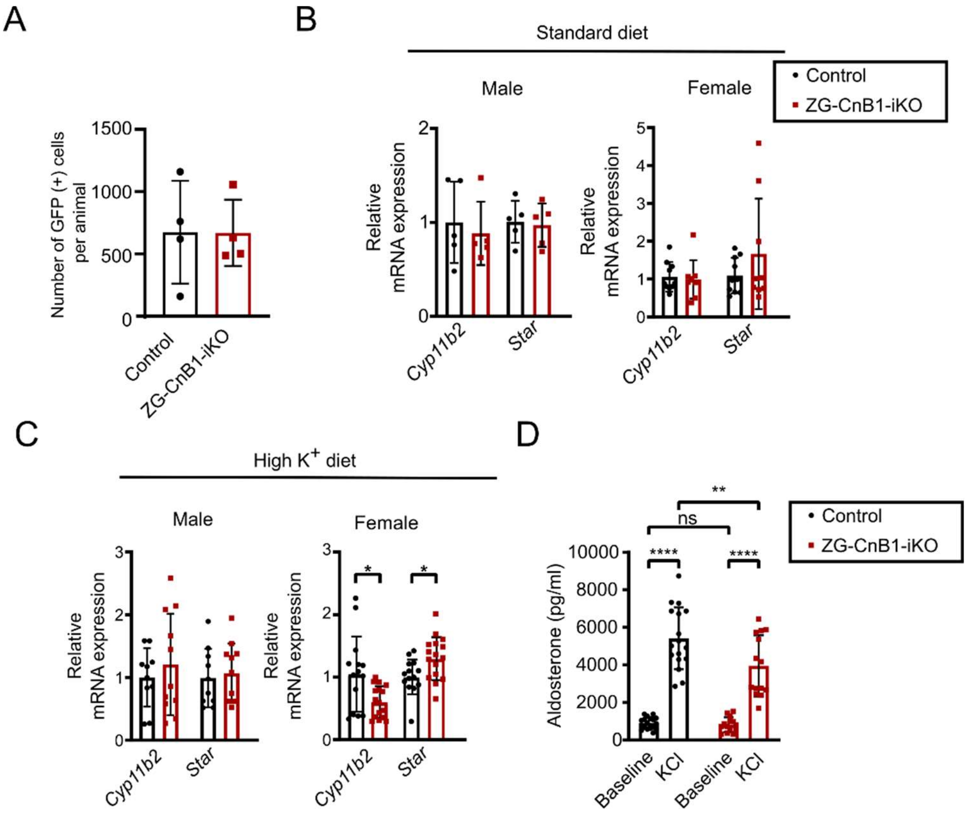

Supplementary Figure 4: Characterization of ZG-CnB1-iKO mice **(A)** Number of GFP (+) cells in adrenals from control and ZG-CnB1-iKO animals determined by fluorescence-activated single cell sorting (FACS). Statistical differences assessed by *Cyp11b2* and *Star* mRNA expression in adrenal gland lysates from control and ZG-Cnb1-iKO animals fed with **(B)** standard and **(C)** high K^+ diet assessed by qPCR. Statistical differences assessed by Student`s t-test. (*p<0.05) **(D)** Ex vivo incubation of adrenal glands from control (n=17) and ZG-CnB1-iKO (n=14) female animals. In the graph, each dot represents the aldosterone production of both adrenals from control or ZG-CnB1-iKO mice. Baseline for each preparation was assessed by measuring aldosterone concentration after incubation with 3 mM K^+. Statistical differences assessed by two-way ANOVA with Tukey`s multiple comparison post-test. (**p<0.01, ****p<0.0001)

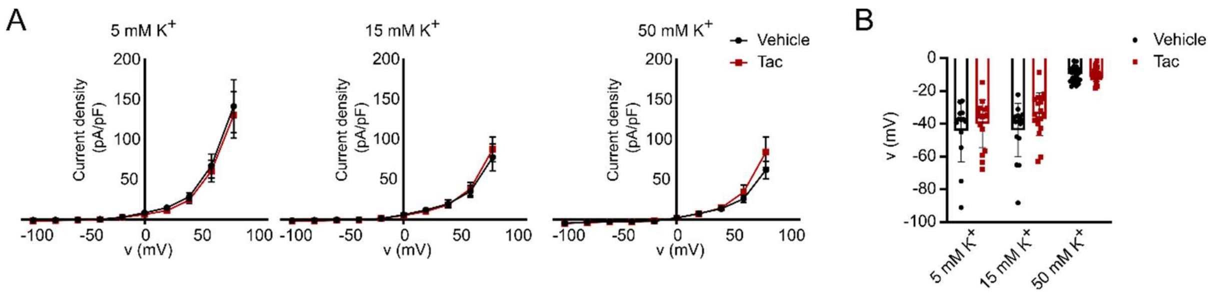

Supplementary Figure 5: Tacrolimus treatment does not affect cell membrane sensitivity to changes in extracellular K^+ concentrations. (A) Current density measured in NCI-H295R cells treated with vehicle or tacrolimus in response to 5 (left panel), 15 (middle panel) and 50 (right panel) mM K^+ in the extracellular medium. (B) Changes in the reversal potential of NCI-H295R cells treated with vehicle or tacrolimus in response to 5-, 15- or 50-mM K^+ in the extracellular medium. Measurements in A and B were assessed in an unbiased manner in freshly detached NCI-H295R cells using automated patch clamp (QPatchII, Sophion).

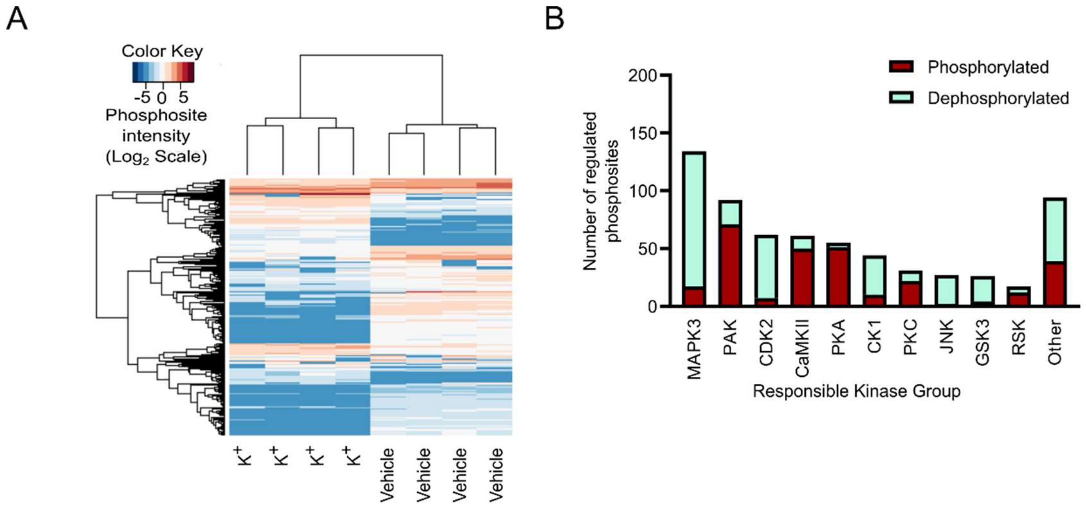

Supplementary Figure 6: Two group comparison of phosphoproteomic data and kinome analysis following K^+ stimulation. **(A)** Heatmap showing unsupervised hierarchical clustering of phosphosites significantly regulated between vehicle and K^+ stimulated cells (qValue < 0.05 and Log2FC > 1). **(B)** The kinome of K^+ stimulated cells was determined by NetworKin analysis using the differentially regulated phosphosites between vehicle and K^+ treatments. The top 10 kinase groups were shown in the graph. (MAPK3: Mitogen activated protein kinase 3, PAK: p21-activated kinase, CDK2: Cyclin dependent kinase 2, PKA: Protein kinase A, CK1: Casein kinase 1, PKC: Protein kinase C, JNK: c-Jun N-terminal kinases, GSK3: Glycogen synthase kinase 3, RSK, Ribosomal S6 kinase)

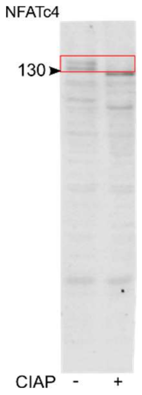

Supplementary Figure 7: Immunoblot profile of NFATc4 is changed upon treatment with alkaline phosphatase. NCI-H295R cell lysates were incubated with calf intestinal alkaline phosphatase (CIAP) for 1 h at 37 °C. Immunoblot showing the total NFATc4 in CIAP-treated NCI-H295R cell lysates. Molecular weight is indicated on the left in kDA. Phospho-specific bands are shown in red rectangle.

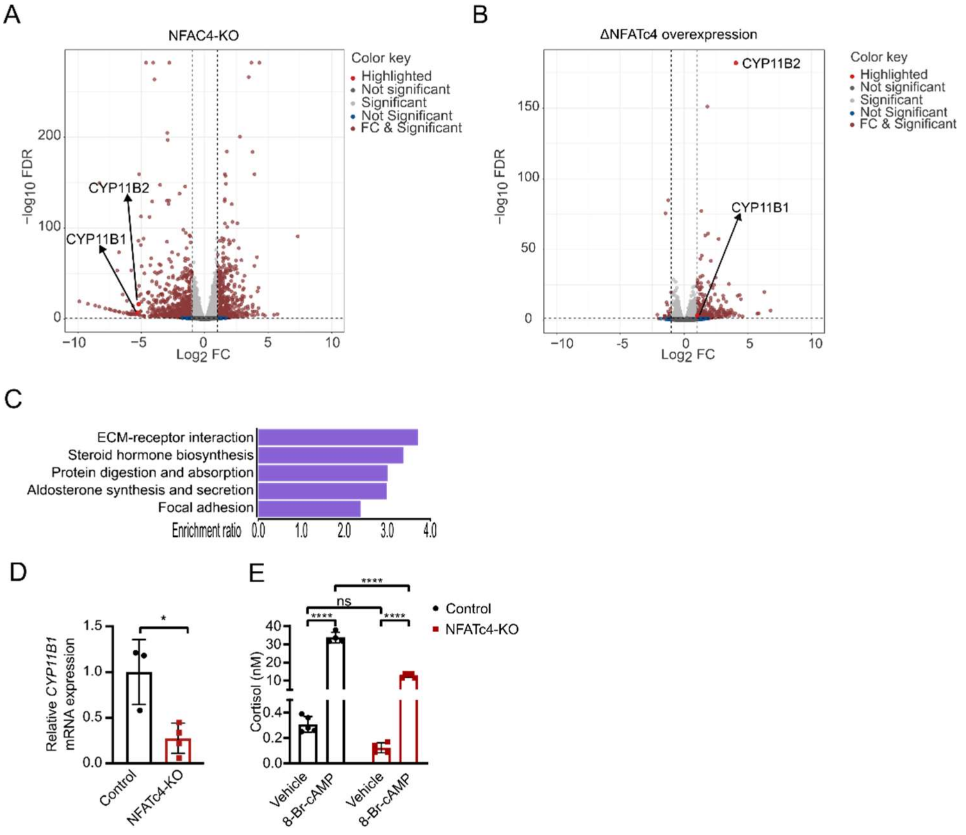

Supplementary Figure 8: Role of NFATc4 in steroidogenesis of NCI-H295R cells. Volcano-plot visualization of differentially expressed transcripts in **(A)** NFATc4-KO NCI-H295R cell line and **(B)** constitutively active NFATc4 (ΔNFATc4) overexpressing NCI-H295R cells. (Log$_2$FoldChange (Log2FC) > 2 and False Discovery Rate (FDR) < 0.05 values are accepted as significant) **(C)** Significantly enriched Kyoto Encyclopedia of Genes and Genomes (KEGG) pathways of dysregulated transcripts in NFATc4-KO NCI-H95R cell line. **(D)** *CYP11B1* mRNA expression in control and NFATc4-KO NCI-295R cells assessed by qPCR. Statistical differences assessed by Student`s t-test. (*p<0.05). **(E)** Cortisol concentration in the supernatant of control and NFATc4-KO was assessed by ultra-performance liquid chromatography tandem-mass spectrometry (UPLC-MS/MS) in cells treated with vehicle, 1 or 0.5 mM 8-bromo-Cyclic AMP (8-Br-AMP) for 48h. Statistical differences assessed by two-way ANOVA with Tukey`s multiple comparison post-test. (ns: not significant, ****p<0.0001)).

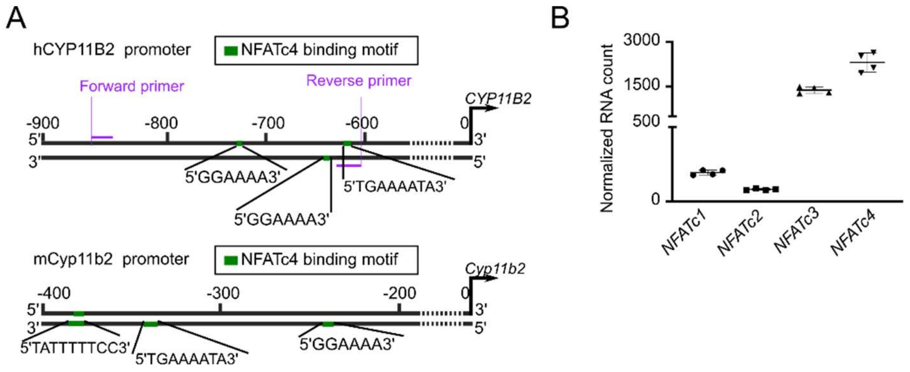

Supplementary Figure 9: NFATc4 binding motifs in CYP11B2 promoter and expression of NFAT isoforms in NCI-H295R cells **(A)** Graphical representation of the predicted NFATc4 binding motifs in human and mouse aldosterone synthase promoter using the PATCH bioinformatic tool. **(B)** mRNA expression pattern of NFATc protein family in control NCI-H295R cell line.

Supplementary Table 1: Patient information of adrenal tissues used ex vivo incubation

Patient #	Sex	Age	Blood K^+ levels before surgery (mM)	Medication
1	Male	59	4	Spironolactone, Amlodipine, Perindopril, Macrogol, Paracetamol, Metamizole
2	Male	53	4.2	Verapamil, Doxazosin, Paracetamol, Metamizole
3	Male	53	4.2	Captopril, Paracetamol, Metamizole
4	Male	64	3.5	Eplerenone, Candesartan, Hydrochlorothiazid, Amlodipin

Supplementary Table 2. Urinary steroid profile of female ZG-CnB1-cKO mice under standard and high K^+ diet measured by UPLC-MS/MS

All values are normalized to urinary creatinine and given as mean ± SD in mg/mg. Statistical differences assessed by Student`s t-test. (*p < 0.05). Number of animals in brackets.

	Standard diet		High K^+ Diet	
	Control (n=4)	ZG-CnB1-cKO (n=6)	Control (n=5)	ZG-CnB1-cKO (n=7)
Aldosterone	1.1 ± 1.2	0.5 ± 0.4	9.9 ± 5.0	3.2 ± 3.0*
18-OH-Corticosterone	94.0 ± 38.5	54.2 ± 22.7	56.0 ± 24.4	32.0 ± 15.1
Corticosterone	29.3 ± 19.0	16.6 ± 6.1	39.5 ± 19.1	38.7 ± 20.7
11-DOC	0.9 ± 0.5	0.9 ± 0.6	2.1 ± 1.0	1.7 ± 0.9
11-dehydrocorti-costerone	7.7 ± 7.5	2.9 ± 1.2	8.0 ± 4.5	5.9 ± 2.8
Progesterone	3.3 ± 2.0	3.0 ± 2.2	8.3 ± 3.3	5.4 ± 2.8
Testosterone	0.3 ± 0.2	0.2 ± 0.1	0.6 ± 0.2	0.4 ± 02

Supplementary Table 3: Blood parameters of ZG-CnB1-iKO mice under standard diet .

All values are given as mean ± SD. Number of animals in brackets.

	Male		Female	
	**Control**	**ZG-CnB1-iKO**	**Control**	**ZG-CnB1-iKO**
pNa^+ (mM)	149.8 ± 0.8 (6)	149.8 ± 0.5 (4)	150.8 ± 0.9 (10)	151.3 ± 1.7 (7)
pK^+ (mM)	4.9 ± 0.3 (6)	4.7 ± 0.3 (4)	4.4 ± 0.3 (10)	4.5 ± 0.5 (7)
pCa^{2+} (mM)	1.3 ± 0 (6)	1.3 ± 0 (4)	1.3 ± 0.1 (10)	1.3 ± 0.1 (7)
pCl^- (mM)	109 ± 0.9 (6)	109 ± 0.8 (4)	110.5 ± 2.5 (10)	111.4 ± 3.8 (7)

Supplementary Table 4: Blood Physiological parameters of ZG-CnB1-iKO mice under high K^+ diet

All values are given as mean ± SD. Statistical differences assessed by Student`s t-test. (*p < 0.05)

	Male		Female	
	**Control**	**ZG-CnB1-iKO**	**Control**	**ZG-CnB1-iKO**
pNa^+ (mM)	154.6 ± 9.7 (9)	151.1 ± 2.2 (12)	150.4 ± 1.5 (14)	149.3 ± 1.1 (15)*
pK^+ (mM)	4.3 ± 0.6 (9)	5.1 ± 2 (12)	4 ± 0.4 (14)	4 ± 0.4 (15)
pCa^{2+} (mM)	1.3 ± 0.1 (9)	1.3 ± 0.1 (12)	1.3 ± 0.1 (14)	1.3 ± 0 (15)
pCl^- (mM)	110.1 ± 2.9 (9)	110.3 ± 3.6 (12)	106.7 ± 3.3 (14)	107 ± 2.8 (15)
pH			7.3 ± 0.1 (14)	7.4 ± 0.0 (15)
Renin *m*RNA (normalized to control)			1.0 ± 1.0 (14)	1.1 ± 0.6 (15)

Supplementary Table 5. Plasma steroid profile of ZG-CnB1-iKO mice under high K+ diet measured by UPLC-MS/MS

All values are given as mean ± SD in nM. Statistical differences assessed by Student`s t-test. (*$p < 0.05$)

	Male		Female	
	**Control**	**ZG-CnB1-iKO**	**Control**	**ZG-CnB1-iKO**
Aldosterone	0.6 ± 0.3 (10)	0.6 ± 0.2 (13)	0.5 ± 0.3 (10)	0.5 ± 0.2 (9)
Corticosterone	252.6 ± 93.3 (10)	287.9 ± 98.2 (13)	214.2 ± 88 (10)	237.6 ± 121.1 (9)
11-DHC	75.5 ± 39.9 (10)	94.2 ± 35.3 (13)	55.1 ± 29.5 (10)	74.7 ± 52.1 (9)
Testosterone	0.3 ± 0.5 (10)	0.6 ± 0.9 (13)	0.1 ± 0.1 (10)	0.1 ± 0.1 (9)
11-DOC	4.6 ± 2 (10)	4.3 ± 2.2 (13)	4.7 ± 1.8 (10)	5.2 ± 1.8 (9)
Androstenedione	0.3 ± 0.1 (10)	0.3 ± 0.2 (13)	0.1 ± 0.1 (10)	0.3 ± 0.1 (9)*
Progesterone			4.4 ± 2.9	5.8 ± 9.8

Supplementary Table 6. Antibody list

Antibody	Host	Source	Catalog number / Reference	Dilution IF	Dilution WB
Anti-pCREB (Ser 133)	Rabbit	Cell Signaling Technology	9198	-	1/1000
Anti-NFATc4	Rabbit	abcam	ab3447	-	1/1000
Anti-Actin	Mouse	Sigma Aldrich	A5316	-	1/5000
Anti-ATF1	Rabbit	Cell Signaling Technology	25177S	-	1/1000
Anti-Tubulin	Rabbit	Cell Signaling Technology	2128	-	1/1000
Anti-rabbit IgG secondary	Goat	Licor	926-32211	-	1/30000
Anti-mouse IgG secondary	Goat	Licor	926-32220	-	1/10000
Anti-Aldosterone Synthase	Rabbit	Dr. Celso E. Gomez-Sanchez (University of Missisipi Medical Center, Jackson, Mississippi, USA)	[Wotus et al. Endocrinology 1998]	1/100	-
Anti-rabbit IgG secondary	Donkey	Jackson Immunoresearch	711-611-152	1/200	-
Anti-rabbit IgG secondary	Chicken	Invitrogen	A-21443	1/200	-
Anti-T7 Tag	Goat	abcam	ab9138	-	-
Polyclonal Goat IgG	Goat	R&D Systems	AB-108-C	-	-

Supplementary Table 7. Primer list

qPCR Primers

Species	Primer Name	Sequence
Human	GAPDH fwd	5`CTGACTTCAACAGCGACACC3`
Human	GAPDH rvr	5`TAGCCAAATTCGTTGTCATAC3`.
Human	CYP11B2 fwd	5`CAGCTGGGACATTGGTACAGG3`
Human	CYP11B2 rvr	5`CAGCGCTGGGGATTATACCG3`
Mouse	mGapdh fwd	5`CCATCACCATCTTCCAGGAG3`
Mouse	mGapdh rvr	5`TCCATGGTGGTGAAGACAC3`
Mouse	mCyp11b2 fwd	5`AACTACAGTGGCATTGTG3`
Mouse	mCyp11b2 rvr	5`GATTGCTGTCGTGTCAAC3`
Mouse	mStar fwd	5`CAGGCATGGCCACACATTTT3`
Mouse	mStar rvr	5`CTGCGATAGGACCTGGTTGA3`
Mouse	mRenin fwd	5`GGAGGAAGTGTTCTCTGTCTACTACA3`
Mouse	mRenin rvr	5`GCTACCTCCTAGCACCACCTC3`

ChIP Primers

Human	CYP11B2 promoter fwd	5`GACCCAGCCTCTAGAAAAAAAA3`
Human	CYP11B2 promoter rvr	5`ATCCTAGGAGTATTTTCACTGTCTT3`

Supplementary Table 8. TaqMan gene expression assay list (Applied biosystems)

Species	Gene	Assay catalog number
Human	GAPDH	Hs02786624_g1
Human	NR4A2	Hs01117527_g1
Human	CYP11B1	Hs01596404_m1
Mouse	Actb	Mm02619580_g1
Mouse	Cyp11b2	Mm01204955_g1
Mouse	Hsd3b6	Mm07306505_m1

Supplementary Methods

Automated Patch clamp

For whole cell automated patch clamp, NCI-H295R cells grown until ~80% confluent and then incubated with vehicle (DMSO) or 10 nM tacrolimus for one hour. Cells were used immediately after detaching with Accutase (Sigma-Aldrich, Buchs, Switzerland) and resuspended in an extracellular solution containing (in mM) 135 NaCl, 1.8 MgCl2, 1.8 CaCl2, 10 HEPES, 5 KCl, pH 7.4 adjusted NaOH / HCl. 3.0 x 106 cells / mL and then directly added to the centrifuge tube of the QPatchII automated patch clamp platform (Sophion Bioscience, Ballerup, Denmark). 48X single hole QChips with a resistance of ~2 MΩ were used for experiments with an intracellular solution containing (in mM) 95 K-gluconate, 30 KCl, 4.8 Na2HPO4, 1.2 NaH2PO4, 5 glucose, 2.38 MgCl2, 0.73 CaCl2, 1 EGTA, 3 Mg-ATP, pH 7.2 adjusted KOH / HCl. No seal enhancer and no junction potential correction (11 mV measured for the solution pair) was used. Only cells maintaining a seal resistance of >1GΩ, and with stable access resistance throughout the experiment, were used (~50% success rate). After obtaining the whole cell patch clamp, the extracellular solution was exchanged twice with solution containing 5, 15, 50 mM K+ and a protocol including 20 mv voltage steps (-100 to +80 mV, holding potential –60 mV, 200 ms) was applied. The osmolality of the extracellular solution was kept constant by proportionally decreasing the concentration of NaCl for increasing concentrations of KCl (e.g., in mM 135 NaCl + 5 KCl or 90 NaCl + 50 KCl) . Current density was calculated using steady state current (average of last 100 ms of each step) divided by the cell capacitance measured immediately before each protocol.

Generation of AS-CreER mouse model

AS-CreER model generation was described in “Leng, Sining. 2019. Regulation of Adrenocortical Morphogenesis and Function by WNT and FGF Signaling. Doctoral dissertation, Harvard University, Graduate School of Arts & Sciences”, accessible at http://nrs.harvard.edu/urn-3:HUL.InstRepos:42029609

Uncropped images of western blots and agarose gel electrophoresis in the manuscript

Full unedited gels for Figure 4

Panel 4A

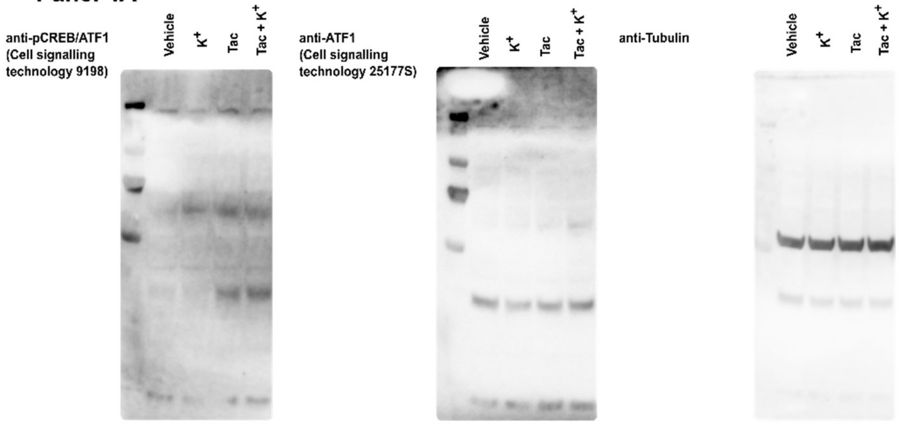

Panel 4F

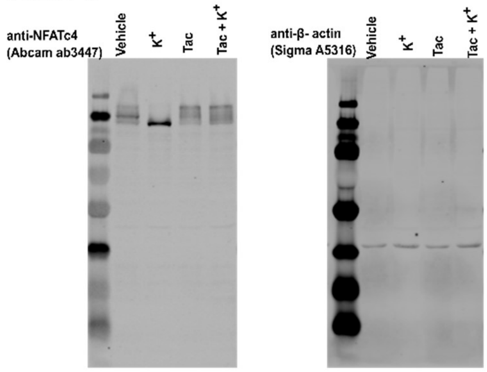

Full unedited gels for Figure 5

Panel 5A

Lanes in black reactangles are shown in the figure.

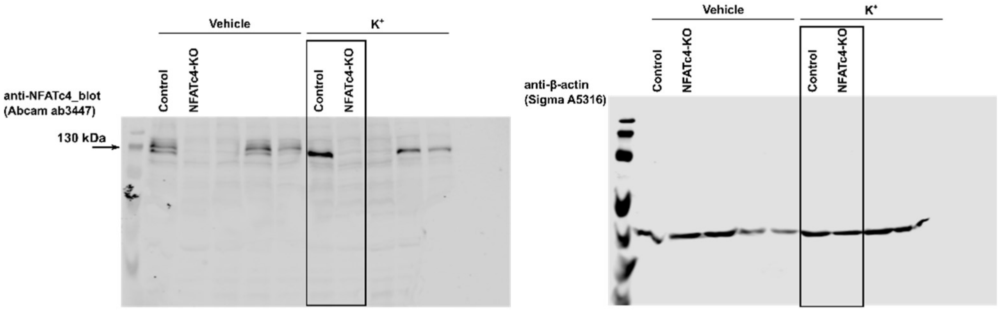

Panel 5F

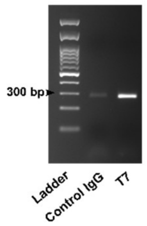

Full unedited gel for Supplementary Figure 7

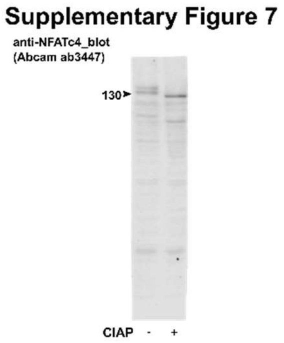

Supplement: Supplemental data [file jciinsight-8-157027-s138.pdf]
